# Coprophagy Prevention Decreases the Reproductive Performance and Granulosa Cell Apoptosis *via* Regulation of CTSB Gene in Rabbits

**DOI:** 10.3389/fphys.2022.926795

**Published:** 2022-07-18

**Authors:** Guohua Song, Yadong Wang, Yaling Wang, Yixuan Jiang, Shuaijie Sun, Hanfang Cai, Guirong Sun, Ming Li, Massimo Bionaz, Huifen Xu

**Affiliations:** ^1^ College of Animal Science and Technology, Henan Agricultural University, Zhengzhou, China; ^2^ Department of Animal and Rangeland Sciences, Oregon State University, Corvallis, OR, United States

**Keywords:** rabbits, coprophagy, reproductive performance, granulosa cells, CTSB

## Abstract

Coprophagy is an instinctive behavior in rabbit with important effects on growth and reproductive performance. The underlying mechanism of this effect in rabbit is unknown. Here, we used Elizabeth circle as a coprophagy preventing model in female rabbits and assess feed intake, growth, and reproductive performance. We found that preventing coprophagy did not affect feed intake but decreased body weight and weight of several organs and tissues and resulted in complete reproductive failure during the late pregnancy period, accompanied by reduced levels of plasma progesterone. RNA-seq analysis of rabbit ovarian tissues revealed that preventing coprophagy affected significantly 241 genes (DEGs), with the large majority being downregulated. Bioinformatic analyses revealed that those DEGs are mostly involved in apoptosis, immune response, and metabolic pathways. Among DEGs, the lysosomal cysteine protease cathepsin B (*CTSB*) was significantly downregulated in the coprophagy prevention group. Further studies using siRNA and adenovirus overexpression systems revealed that CTSB promotes the proliferation of rabbit granulosa cells (GCS) and prevents apoptosis. Measurement of transcripts coding for proteins related to apoptosis revealed a minor transcriptomic effect of CTSB, indicating that its effect is likely post-transcriptional. Overexpression of CTSB increased secretion of progesterone and estradiol, partly *via* upregulation of *CYP19A1* while inhibition of CTSB decreased progesterone secretion partly *via* downregulation of the StAR gene. In conclusion, our study demonstrated the detrimental effect on reproduction by preventing coprophagy with a main role for this response played by CTSB on the granulosa cells of the ovary.

## Introduction

Coprophagy is a terminology that is used to describe the behavior of eating feces in small herbivores (Garcia et al., 2004; Ferreira et al., 2018). Two types of coprophagy behavior have been observed: the ingestion of own feces (autocoprophagy) or ingestion of excrements of another animal of the same species (allocoprophagy) (Galef and Bennett, 1979). This behavior allows hindgut-fermenting mammals to recover the nutrients that were liberated in the hindgut and aids the absorption of essential amino acids, vitamin B, vitamin K, and trace elements, thereby avoiding the loss of these nutrients (Savage, 1986). Due to the colonic separation mechanism, many small herbivores such as rabbits, golden hamsters, rodents, and koalas produce two types of feces (hard feces and soft feces), and they acquire the behavior of coprophagy during specific growing periods or under conditions of malnutrition (Sakaguchi, 2003).

The nutritional importance of coprophagy has been demonstrated, and it can increase the total intake of protein up to 38% in growing rabbits (Gidenne, 2000; Belenguer et al., 2005; Liu et al., 2007). Coprophagy can avoid the loss of endogenous nitrogen and increase absorption of essential amino acids, providing up to 23% of lysine for lactating rabbits (Abecia et al., 2008; García et al., 2004). In addition to nutritional benefits, coprophagy behavior is helpful for maintaining gut microflora homeostasis and this was supported by the evidence that fasting coprophagy resulted in marked changes in the diversity of caecal microorganisms (Klaasen et al., 2009; Kobayashi et al., 2019; Li et al., 2020), affecting nutrition and energy homeostasis (Tremaroli and Backhed, 2012; Zietak et al., 2016). The maintenance of the gut microbiota by coprophagy is also important for cognitive behavior, as observed in Brandt’s vole (Bo et al., 2020).

Coprophagy behavior in small herbivores is an adaptive strategy to the disadvantage of the small body size for the digestion of low-quality fiber (Pei et al., 2001; Liu and Wang, 2007; Sakamaki, 2010). Coprophagy has great impact on the growth of rabbits, as the body size and body weight are significantly decreased when coprophagy is prevented (Sukemori et al., 2006; Wang et al., 2019; Bo et al., 2020). Coprophagy is also important for pregnancy and lactation, as female mice exhibit coprophagy more frequently and ingest more feces during pregnancy and lactation than the non-pregnant period (Ebino et al., 1988). Prevention of coprophagy in mice increases abortion, likely due to deficiency of vitamin B12 and folic acid during pregnancy (Sukemori et al., 2006; Ebino et al., 1989).

The aforementioned data indicated that coprophagy is an important behavior during pregnancy in mice; however, the role of coprophagy on reproduction of rabbits has not been studied. Our hypothesis is that coprophagy positively affects reproduction in rabbits. To assess our hypothesis, we used Elizabeth circle to prevent coprophagy for 5 months in female rabbits and assessed its effects on growth and reproductive performance. Furthermore, *via* RNA-seq of ovary and molecular biology approaches using cumulus cells, we attempted to reveal the molecular mechanism responsible for the reproductive response mediated by coprophagy in rabbits.

## Material and Methods

### Ethic Statement

The present study was approved by the Institutional Animals Care and Use Committee (IACUC) of the College of Animal Science and Technology of Henan Agricultural University, China (Permit Number: 11-0085; Data: 06-2011) and was designed and performed according to the institutional guidelines.

### Animals

For the experiment we used 12 healthy, weaned 30-days old female New Zealand white rabbits with similar body weight (1.14 ± 0.12 kg) from the animal experimental center of Henan Agricultural University. The rabbits were maintained in individual cages and were randomly divided into two groups: control group and experimental group (i.e., coprophagy prevention group). The coprophagy prevention group was fitted with a 7-cm wide Elizabeth circle to prevent coprophagy (wang et al., 2019). Food intake of the rabbits was measured every day. Body weight of the rabbits was measured every week. After 22 weeks of feeding, rabbits received an intramuscular injection of chorionic gonadotrophin (SanSheng Biotechnology, NingBo, China), followed by artificial insemination (210 days). Pregnancy diagnosis was performed by using B-ultrasound (Mindary, DP-10) 15 days after artificial insemination (225 days).

### Sample Collection

After giving birth to pups and/or on the 30th day of pregnancy, rabbits were anesthetized followed by euthanization for the collection of samples. The abdominal fat, liver, stomach, kidney, uterus, lung, heart, and spleen were collected and weighed. All tissue samples, including ovaries, were flash-frozen in liquid nitrogen and preserved at -80°C. The blood samples were collected and placed in pro-coagulant tubes (Shandong Ao Sai Te Medical Devices Co’ LTD, Shandong, China) during sacrifice, the serum was obtained by centrifuging and stored at -80°C for analysis.

### Analysis of Blood Biochemical Parameters

All kits used to measure serum biochemical parameters were purchased from the Nanjing Jiancheng Institute of Biological Engineering (Nanjing, China) and measured according to the manufacturer’s instructions using a microplate reader (Bio-Rad). The parameters examined included high-density lipoprotein cholesterol (Cat. No.F003-1-1), low-density lipoprotein cholesterol (Cat. No. A019-2-1), total cholesterol (Cat. No. F002-1-1), triglyceride (Cat. No. A110-1-1), albumin (Cat. No. A028-1-1), and progesterone (Cat. No. H089).

### RNA Extraction and cDNA Library Construction

Total RNA of ovary samples was extracted by using TRIzol reagent (Invitrogen, CA, United States) and stored at -80°C. The quality and concentration of the total RNA was quantified by using the Nanodrop 2000 spectrophotometer (Thermo Scientific). RNA integrity was measured using the Nano 6000 LabChip kit (Agilent, Technologies, CA, United States). The RIN (RNA integrity number) of all RNA samples was >8.6. One aliquot of the RNA per each sample (n = 3 per group) was sent for the preparation of RNA-seq libraries (Biomarker Biotechnology Co., Ltd, Beijing, China), and the remaining aliquot of RNA samples was used for RT-qPCR analysis.

### Transcriptome Library Construction and Sequencing

The library of each sample was sequenced using Illumina HiSeqTM 2500 (Biomarker Biotechnology Co., Ltd, Beijing, China). The Q20, Q30, GC-content, and sequence duplication level of the clean data were calculated, the clean data (clean reads) were obtained by removing reads containing adapter, reads containing ploy-N, and low-quality reads from raw data.

### Reads Alignment and Differential Expression Analysis for RNA-Seq

Clean reads were mapped to the reference genome of the rabbit (Orycun2.0) and annotated using TopHat 2 software (http://ccb.jhu.edu/software/tophat/index.shtml). Cufflinks software (Cufflinks, Berkeley, CA, United States) was used for splicing and expression quantification. The dataset is available in the public repository GEO NCBI Sequence Read Archive (SRA accession PRJNA767495).

Statistical analysis of data normalized by the RPKM method (reads per kilo base transcriptome per million mapped reads) was performed using the DESeq R package (1.10.1) (Anders and Huber, 2010), with negative binomial distribution. The resulting *p*-value was adjusted using Benjamin and Hochberg’s multiple testing correction for controlling the false discovery rate (FDR). An FDR <0.05 was considered as the thresholds for screening significantly differentially expressed mRNA (DEGs).

### Downstream Bioinformatic Analyses

Bioinformatics analysis was performed using three different tools. The KEGG database was used for the DEGs pathway analysis using KOBAS (http://kobas.cbi.pku.edu.cn/) (Mao et al., 2005). Pathways with a Q value ≤0.05 were defined as significantly enriched. The data were analyzed also using the Dynamic Impact Approach (DIA) (Bionaz et al., 2012) and Database for Annotation, Visualization, and Integrated Discovery (DAVID) (Sherman et al., 2022). For the DIA, the orthologous mouse gene IDs were used after annotation of the rabbit gene IDs were performed using the biological DataBase network (bioDBnet) (Mudunuri et al., 2009). The whole annotated gene IDs were used as background for both DAVID (where the Ensembl gene IDs were used) and DIA (where the Entrez Gene IDs were used). For DAVID, an EASE score ≤0.05 was used to identify significant enriched terms, and results were downloaded as a functional annotation chart, using the default DAVID annotation categories. The Ensembl gene IDs with an FDR<0.05 were uploaded into DAVID as all DEGs, upregulated DEGs, and downregulated DEGs.

### Separation and Culture of Rabbits GCS

The rabbits’ granulosa cells (GCS) were isolated and cultured as previously described (Song et al., 2021) from the ovaries of three rabbits purchased from the Laboratory Animal Center (Zhengzhou, Henan, China). Briefly, ovaries from 6 months female rabbits were collected and washed in PBS (with 1% penicillin/streptomycin, 37°C). These rabbits were first intramuscularly injected with 50 IU pregnant mare serum gonadotropin (Solarbio, Beijing, China) to induce estrus. The follicles were punctured with 1 ml syringe needle to collect GCS. Those were cultured in a basal culture medium which consisted of DMEM/F12 medium (Gibco, United States) supplemented with 15% fetal bovine serum (Gibco, United States) and 1% penicillin/streptomycin (Hyclone, United States). The GCS were collected by centrifugation (5 min, 1,000 rpm/min) and incubated in basal culture medium at 37°C in 5% CO_2_, and cell medium was changed every 24 h.

### Immunostaining

Triplicate for each rabbit of sections of ovarian tissue and GCS-crawling film were used for immunofluorescence staining. Ovarian tissue sections and GCS-crawling film were fixed with 4% paraformaldehyde for 15 min and then permeabilized with 0.1% Triton X-100 for 10 min. Then cell-climbing slices were incubated with an antifollicle-stimulating hormone receptor (MAB65591, 1: 200, R&D Systems, Minneapolis, United States) and ovarian sections were incubated with anti-CTSB (48118-1, 1:300, Signalway Antibody, Maryland, United States) rabbit polyclonal antibody for 1 h at 37°C. The sections were then incubated with allophycoerythrin (APC)-goat antirabbit Ig G (GB25303, 1:400, Servicebio, Wuhan, China) at 37°C for 45 min. Finally, the ovarian section and cell-climbing slices were taken out and mounted with DAPI medium (Solarbio). The results were observed under a laser scanning confocal microscope (Nikon Eclipse C1).

### TUNEL Assay

The ovaries were made into paraffin sections, deparaffinized, and rehydrated. The ovaries were incubated with proteinase K working solution at 37°C for 25 min, and covered with permeabilization working solution (Servicebio; Hubei, China) for 20 min. According to the instructions of the TUNEL assay kit (Roche; Basel, Switzerland), the ovaries were incubated with mix reagent 1 (TdT) and reagent 2 (dUTP) at 37°C for 2 h. Cell nuclei were stained with DAPI (Beyotime). The cells were observed under a fluorescence microscope (Nikon, Tokyo, Japan).

### HE Staining

GCS were seeded in 6-well culture plates with cell-climbing slices. The cells were fixed with 95% ethanol for 20 min and stained with hematoxylin dye solution for 3 min. After dehydration with gradient alcohol, the cells were dyed with eosin dye solution for 5 min. Finally, the cells were mounted with neutral gum and observed under a microscope (Nikon Eclipse E100).

### siRNA Interference

The specific siRNA sequence targeting CTSB was designed and synthesized by GenePharma (Shanghai, China). CTSB-siRNA, sense: 5′-CCG​GGC​ACA​ACU​UCU​UCA​ATT-3′ and antisense: 5′-UUG​AAG​AAG​UUG​UGC​CCG​GTT-3′. NC-siRNA, sense: 5′-UUC​UCC​GAA​CGU​GUC​ACG​UTT-3′ and antisense: 5′-ACG​UGA​CAC​GUU​CGG​AGA​ATT-3′. siRNA was transfected into GCS by using Lipofectamine 3000 (Invitrogen, Carlsbad, CA, United States), according to the manufacturer’s instructions.

### Recombinant Adenoviruses Generation

The production of recombinant adenovirus was previously described (Song et al., 2021). Briefly, CTSB gene was amplified and subcloned into the shuttle vector pAdTrack-CMV. The linearized product of pAdTrack-CMV-CTSB was transformed into *Escherichia coli* BJ 5183 competent cells containing the backbone vector pAdEasy-1 to obtain the positive recombinants of pAd-CTSB. Linearized pAd-CTSB fragment was transfected into HEK293 cells for adenovirus packaging and amplification. GCS were infected with pAd-CTSB at an MOI value of 40.

### RT-qPCR

Total RNA of ovary tissues and rabbit GCS was extracted with the TRIzol reagent (Invitrogen, Carlsbad, CA, United States) and RNAprep pure cell kit (Tiangen Biotech Co. Ltd., Beijing, China), respectively. cDNA was synthesized using PrimeScript RT kit (Takara, Tokyo, Japan). RT-qPCR was performed using SYBR®Premix Ex TaqTM ⅡKit (Takara, Tokyo, Japan) on a CFX96TM real-time PCR detection system (Bio-Rad Laboratories Inc, Hercules, CA). All the primer sequences were designed by using NCBI (https://www.ncbi.nlm.nih.gov/) and Oligo 7 software (Molecular Biology Insights, Cascade, CO) and are listed in Supplementary Table S1. LinRegPCR was used to calculate the final RT-qPCR data (Ruijter et al., 2009). Four internal control genes were tested (*GAPDH*, *YWHAZ*, *ACTB*, and *HPRT1*). According to geNorm results, the geometrical mean of three reference genes (*YWHAZ*, *ACTB*, and *HPRT1*) was used to normalize RT-qPCR for the ovary transcriptome and the geometrical mean of *ACTB* and *HPRT1* for the *CTSB* overexpression or inhibition experiments (see Supplementary Figure S3). Final data for each sample were calculated as expression ratio relative to the mean of the control group in all experiments.

### Western Blot

After 48 h treatment with recombinant CTSB adenovirus or siRNA, rabbit GCS cultured in 6-well culture plates were washed with PBS and lysed using RIPA lysis buffer (Epizyme, Shanghai, China). Protein concentration was quantified with a BCA kit (Beijing, China), processed with 12.5% sodium dodecyl sulfate-polyacrylamide gel electrophoresis, and translocated to nitrocellulose membranes. The membrane was sealed with 5% skimmed milk for 2 h and incubated with anti-CTSB (48118-1, 1:5000, Signalway Antibody, Maryland, United States) and β-actin (GB12001, 1:1000, Servicebio, Wuhan, China) at 4°C overnight followed by incubation with goat antirabbit IgG antibody (SA00001-1, 1:1000, Proteintech, Wuhan, China) at room temperature for 2 h. Blots were visualized using GE Amersham Imager 600 (General Electric Company, Bosto, MA, United States) and analyzed with ImageJ.

### Progesterone and Estradiol

Rabbits GCS were cultured in a 6-well culture plate, after 48 h treatment with recombinant CTSB adenovirus and siRNA, and the culture medium was collected for hormone detection. Progesterone and estradiol were quantified with commercial kits, according to the manufacturer’s instructions (Ruixin, Quanzhou, China). The absorbance was measured using a microplate reader (Bio-Rad) at 450 nm.

### Cell Viability Assay

GCS (1×10^4^/well) were seeded in 96-well plates. After treatment with recombinant CTSB adenovirus and siRNA for 12, 24, 36, and 48 h, GCS were collected for proliferation assay by Cell Counting Kit-8 (CCK8) (US Everbright^®^ Inc., Jiangsu, China). Ten μL of CCK8 solution was added to each well. After 2 h incubation, the absorbance was measured by a microplate reader (Bio-Rad) at 450 nm.

### Cell Apoptosis Analysis

Rabbits GCS were cultured in a 12-well culture plate, after treatment of GCS for 48 h treatment with recombinant CTSB adenovirus or siRNA, cell apoptosis was detected with the Annexin V-Alexa Fluor 647/7-AAD apoptosis kit (4A Biotech., Beijing, China). In short, GCS were digested with 0.05% trypsin (without EDTA) and resuspended with cold PBS, and then mixed with 5 μl Annexin V/Alexa Fluor 647 at room temperature for 5 min. Finally, 10 μl 7-AAD and 400 μl PBS were added. The apoptosis rate of GCS was measured with a CytoFLEX flow cytometer (Beckman CytoFlex, CA, United States).

### Statistical Analysis

All treatments were performed in biological triplicate, and data are presented as means ± SE. Statistical analysis for all parameters except RT-qPCR data were performed with one-way ANOVA to compare the effects of different treatments by SPSS statistics 22.0 (SPSS, Chicago, IL, United States). For RT-qPCR data, a general linear model (SAS v9; SAS Institute, Cary, NC, United States) with treatment (coprophagy prevention or control; overexpression of CTSB or wild-type; and interference CTSB or wild-type) as main effect was used. Differences were considered statistically significant when *p* < 0.05.

## Results

### Preventing Coprophagy Decreases Growth in Rabbits

**FIGURE 1:**
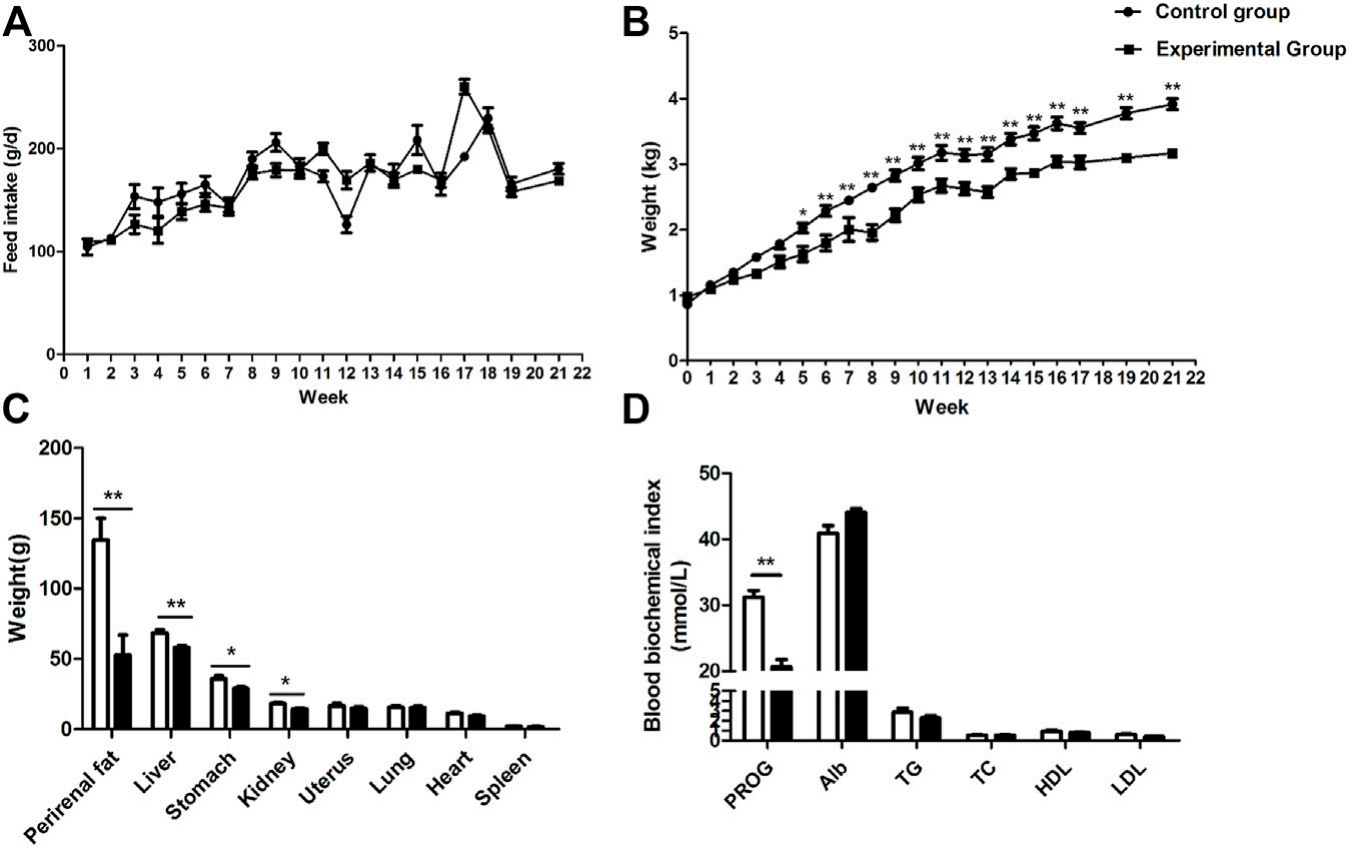
Effects of coprophagy prevention on **(A)** feed intakes; **(B)** body weight; **(C)** weight of different tissues and internal organs; and **(D)** serum biochemical parameters in rabbits [progesterone (PROG), albumin (Alb), triglycerides (TG), total cholesterol (TC), HDL-cholesterol (HDL), and LDL-cholesterol (LDL)]. Statistical differences between groups were indicated as * for *p* < 0.05, ** for *p* < 0.01, and *** for *p* < 0.001.

Prevention of coprophagy did not affect feed intake (Figure 1A, *p* = 0.53) but decreased body weight of the rabbits from the fifth week to the end of the experiment (Figure 1B, *p <* 0.05). The weight of the liver, perirenal fat, stomach, and kidney in the coprophagy prevention group was significantly lower than the control group (Figure 1C, *p <* 0.05). No differences were observed in the levels of serum albumin, triglycerides, total cholesterol, HDL-cholesterol, and LDL-cholesterol between the control group and the coprophagy prevention group (Figure 1D, *p* > 0.05). However, serum progesterone was significantly decreased by preventing coprophagy (Figure 1D, *p <* 0.05).

### Preventing Coprophagy Reduces Reproduction Performance

Fifteen days after artificial insemination, rabbits of the two groups were subjected to B-mode ultrasound examination to check the status of pregnancy of the animals. As shown in Supplementary Figure S1, compared with non-pregnant rabbits, embryos in the rabbits of the control group and coprophagy prevention group were clearly detected, indicating that rabbits in both groups were all pregnant. However, after 30 days of pregnancy, only the rabbits in the control group gave birth to newborn rabbits, while rabbits in the coprophagy prevention group did not gave birth to any newborn rabbits (Table 1). Despite the lack of any litter, we did not observe dead fetuses in the uterus after slaughter that happened just after the due date.

**TABLE 1:** Reproduction performance of the rabbits.

Item	Treatment		*p*-Value
	Control	Experimental	
Total litter size born per doe	5.50 ± 0.57	0.00 ± 0.00	<0.01
Birth weight of litters (g)	54.5 ± 5.89	0.00 ± 0.00	<0.01
Rabbits born alive per doe	5.25 ± 0.96	0.00 ± 0.00	<0.01
Survival ratio of the newborn rabbits (%)	0.95 ± 0.10	0.00 ± 0.00	<0.01

### Coprophagy Prevention Increased the Apoptosis of Ovarian Tissue Cells

**FIGURE 2:**
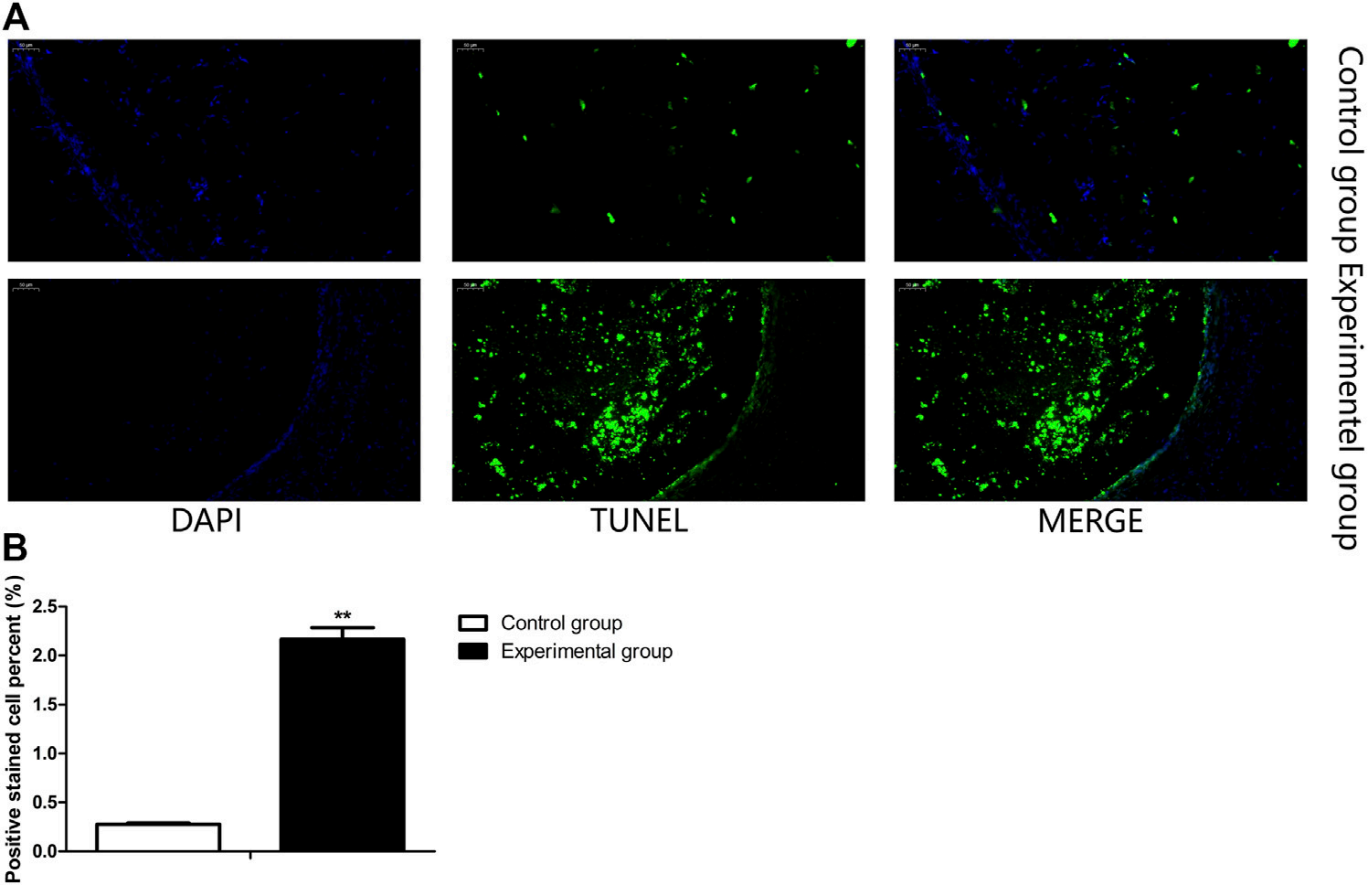
Coprophagy prevention increased cell apoptosis in ovary of rabbits. **(A)** Representative images of ovarian stained by TUNEL assay, cell nuclei were stained by DAPI (blue), apoptotic cells were stained with TUNEL (green). **(B)** Quantification of the proportion of ovarian apoptotic cells as detected by TUNEL assay in each group of rabbits. Scale bar = 50 μm **p < 0.01.

In order to investigate the possible mechanism of coprophagy prevention on reproduction, TUNEL assay was performed to measure the apoptosis of ovarian tissue cells. As shown in Figure 2, the proportion of apoptotic cells was significantly higher in the ovarian tissue of rabbits where coprophagy was prevented compared to the control group.

### Effects of Coprophagy Prevention on the Ovarian Transcriptome

**FIGURE 3:**
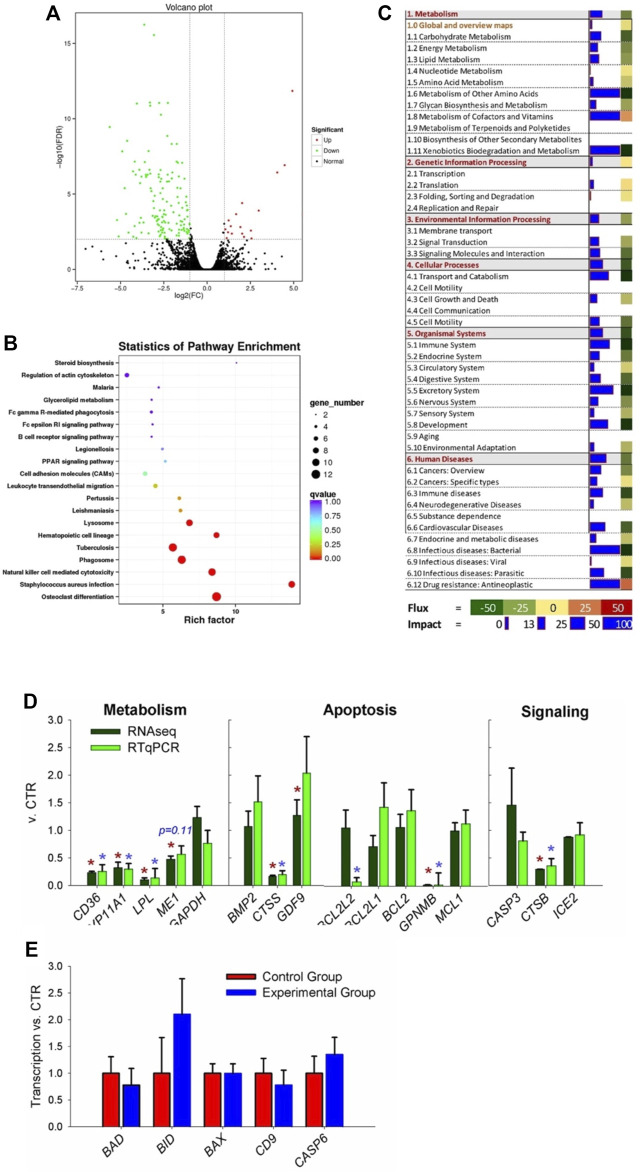
Transcriptomic analysis of ovary tissues in rabbit where coprophagy was prevented (i.e., experimental group) and a control group (n = 3 per group). **(A)** Volcano plot of differentially expressed genes (expression ratio experimental/control group). **(B)** KEGG pathways enriched (*p* < 0.05) in DEGs by using KOBAS. **(C)** Summary of main categories of KEGG pathways generated by using the Dynamic Impact Approach. Blue horizontal bars denote impact (larger the bar, larger the impact) and squares besides the blue horizontal bars denote flux of the pathway that can be induced or inhibited as indicated in the bottom legend. **(D)** RNA-seq vs. RT-qPCR for transcripts selected among the ones related to metabolism, apoptosis, and signaling pathways. **(E)** Additional transcripts measured by RT-qPCR involved in apoptosis and related to cathepsin B activity.

A total of 14,378 transcripts with a minimum count of four in at the least two samples were expressed in ovaries of the two groups of rabbits, 241 DEGs were present in these two groups, in which 210 were downregulated and 31 were upregulated DEGs (Figure 3A, FDR ≤0.05; complete dataset is available in Supplementary File S1).

Bioinformatics analysis of DEGs performed using KOBAS not only revealed an enrichment of KEGG pathways related to differentiation (specifically of osteoclasts and hematopoietic cells), infection, and immune response (e.g., response to *Staphylococcus* infection, natural killer cell-mediated cytotoxicity, leukocytes migration, and several human-related diseases) but also pathways related to apoptosis, such as ‘Lysosome’ and ‘Phagosome’ (Figure 3B). Few KEGG pathways related to metabolism were significantly enriched, including PPAR signaling, glycerol-lipid metabolism, and steroid biosynthesis.

Summary of KEGG pathway as performed by DIA (Figure 3C), revealed an overall inhibition on all categories of pathways except ‘genetic information processing.’ Metabolism of other amino acids, cofactors, vitamins, and xenobiotic were among the most impacted and inhibited metabolic sub-category of pathways. When looking at the detail of each metabolic pathway, ‘nicotinate and nicotinamide metabolism’ was highly activated and ‘folate biosynthesis,’ ‘drug metabolism–cytochrome P450,’ and ‘glycolipid metabolism’ were highly inhibited (Supplementary File S2). Several signaling pathways were impacted by the prevention of coprophagy on ovaries, such as ‘PPAR signaling’ and ‘cytokine–cytokine receptor interaction’ that were inhibited and ‘Wnt signaling pathway’ that was activated (Supplementary File S2). The pathways associated with apoptosis were overall inhibited as well pathways associated with the immune system, such as ‘natural killer cell mediated cytotoxicity,’ ‘antigen processing and presentation,’ and ‘Toll-like receptor signaling pathway.’ Highly inhibited pathways were also associated with transport, catabolism, and apoptosis, such as ‘lysosome,’ ‘endocytosis,’ ‘phagosome,’ and ‘apoptosis’ (Supplementary File S2). Among pathways associated with diseases, the most impacted and inhibited was ‘*Staphylococcus aureus* infection,’ ‘tuberculosis,’ and ‘chemical carcinogenesis’ (Supplementary File S2).

Results from DAVID were somewhat consistent with the other bioinformatics tools. Highly enriched were pathways associated with disease and immune response (e.g., ‘*Staphylococcus aureus* infection’ and ‘natural killer cell mediated cytotoxicity’), lipid metabolism (e.g., ‘glycerolipid metabolism’), signaling (e.g., ‘JAK-STAT signaling pathway’), and organelles involved with apoptosis (e.g., ‘lysosome’ and ‘phagosome’) (Supplementary File S2). When considering additional annotations, DAVID results revealed significant enrichment of membrane-associated components, immunoglobulins, diseases-associated pathways, glycoproteins, peptidases associated with the lysosome, and lipid metabolism-related terms. All the enriched terms in DAVID were downregulated DEGs (Supplementary File S2).

We selected 16 transcripts coding for proteins related to metabolism, apoptosis, and signaling to validate the RNA-seq data (Figure 3D). Except for 1 gene (*BCL2L2*), all of the other transcripts had the same pattern and for 14 out 16 transcripts the statistical effect was conserved.

### Role of Cathepsin B in Response to Coprophagy in Ovaries

Among the transcripts selected for validation, the cathepsin B (*CTSB*) was significantly decreased by the prevention of coprophagy (Figure 3D). The reasons for focusing on this transcript are: 1) the *CTSB* transcript codes for a member of the cathepsin family, which is mainly expressed in lysosomes and plays a variety of roles in antigen presentation (Brix et al., 2008), apoptosis (Conus and Simon, 2010), autophagy (Tardy et al., 2006), and cell homeostasis (Li and Albertini, 2013), all functions and terms that were revealed to be important by bioinformatics results of the transcriptomic data in the ovary; 2) CTSB plays an important role in cell autophagy and apoptosis (Morchang et al., 2013; Tatti et al., 2013), which could help explaining the high apoptosis we observed in ovaries when rabbit were prevented to perform coprophagy. It has been reported that CTSB plays an important role in regulating the formation of cumulus-oocyte complex and the maturation of oocytes, which will subsequently affect reproductive performance (Balboula et al., 2010; Balboula et al., 2013). However, the role of the CTSB gene in rabbit reproductive performance has not been previously studied.

CTSB in the cytoplasm initiates the lysosomal pathway of apoptosis, mainly through cleavage of BH3-interacting domain death agonist (BID) and anti-apoptotic degradation of BCL-2 homologues (Repnik et al., 2012). Thus, to further evaluate if CTSB is implied in regulating apoptosis in the ovaries of rabbit *via* the BCL-2 pathway, we performed RT-qPCR analysis of additional transcripts that were not in the RNA-seq dataset but are known to be important in apoptosis, such as BCL2-associated X (BAX), BID and BCL2-associated agonist of cell death (BAD) (Figure 3E). We were able to detect all those transcripts but none resulted to be differentially expressed in the ovary of the two groups.

### Isolation and Identification of Rabbit Ovarian Granular Cells

**FIGURE 4:**
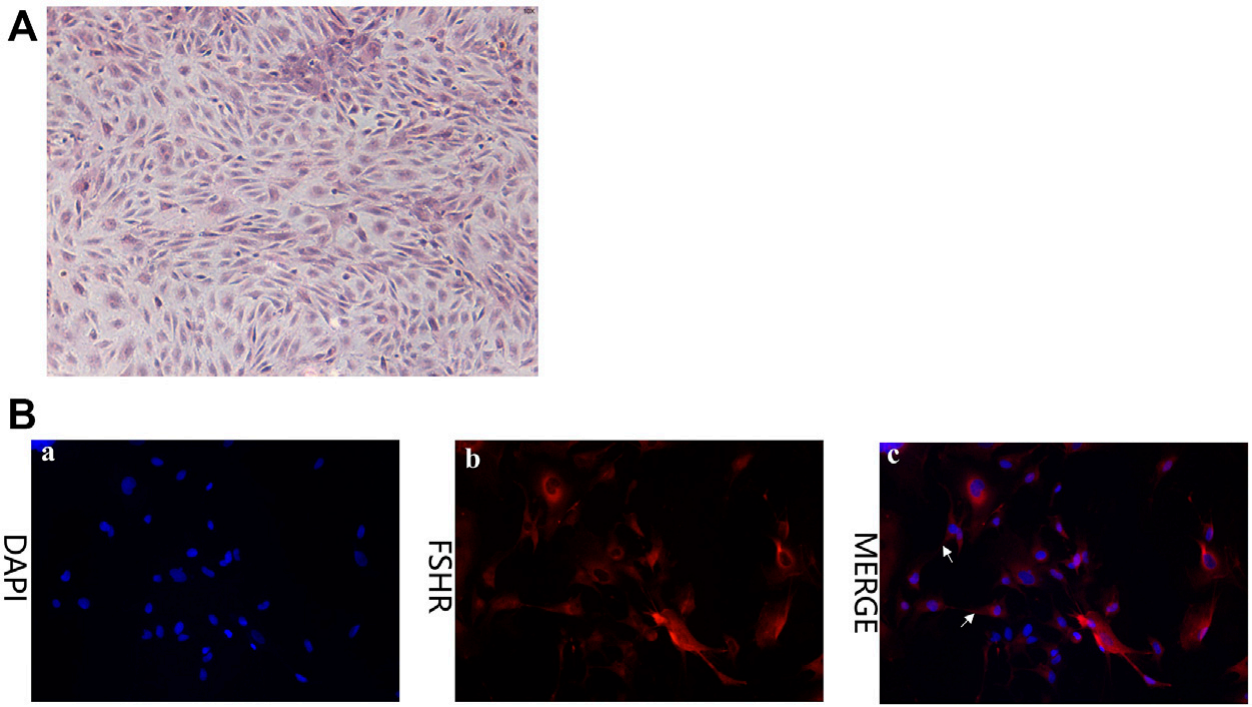
Characterization of granulosa cells (GCS) isolate from ovaries of healthy female rabbits (5 months of age). **(A)** H&E staining (40×). **(B)** Expression of follicle-stimulating hormone receptor (FSHR) in GCS isolated from ovaries of rabbits as detected *via* immunofluorescence staining (red = FSHR; blue = nuclei) (200×). Pseudopodia between cells are indicated with white arrows.

In order to investigate the role of CTSB in reproduction and the association between coprophagy prevention and reproductive failure, GCS of rabbits were isolated from three female rabbits and stained with hematoxylin and eosin (H&E) (Figure 4A). The cells exhibited a typical morphology expected for GCS, with obvious nuclei (blue) and cytoplasm (red) and with pseudopodia between the cells closely connected. Identification of rabbits GCS was performed by using immunofluorescence staining with the marker protein follicle-stimulating hormone receptor (FSHR). As shown in Figure 4B, FSHR protein was abundantly present in the cytoplasm of the cells, indicating that the isolated cells were indeed GCS.

### Efficiency of CTSB Overexpression and siRNA Interference in GCS

**FIGURE 5:**
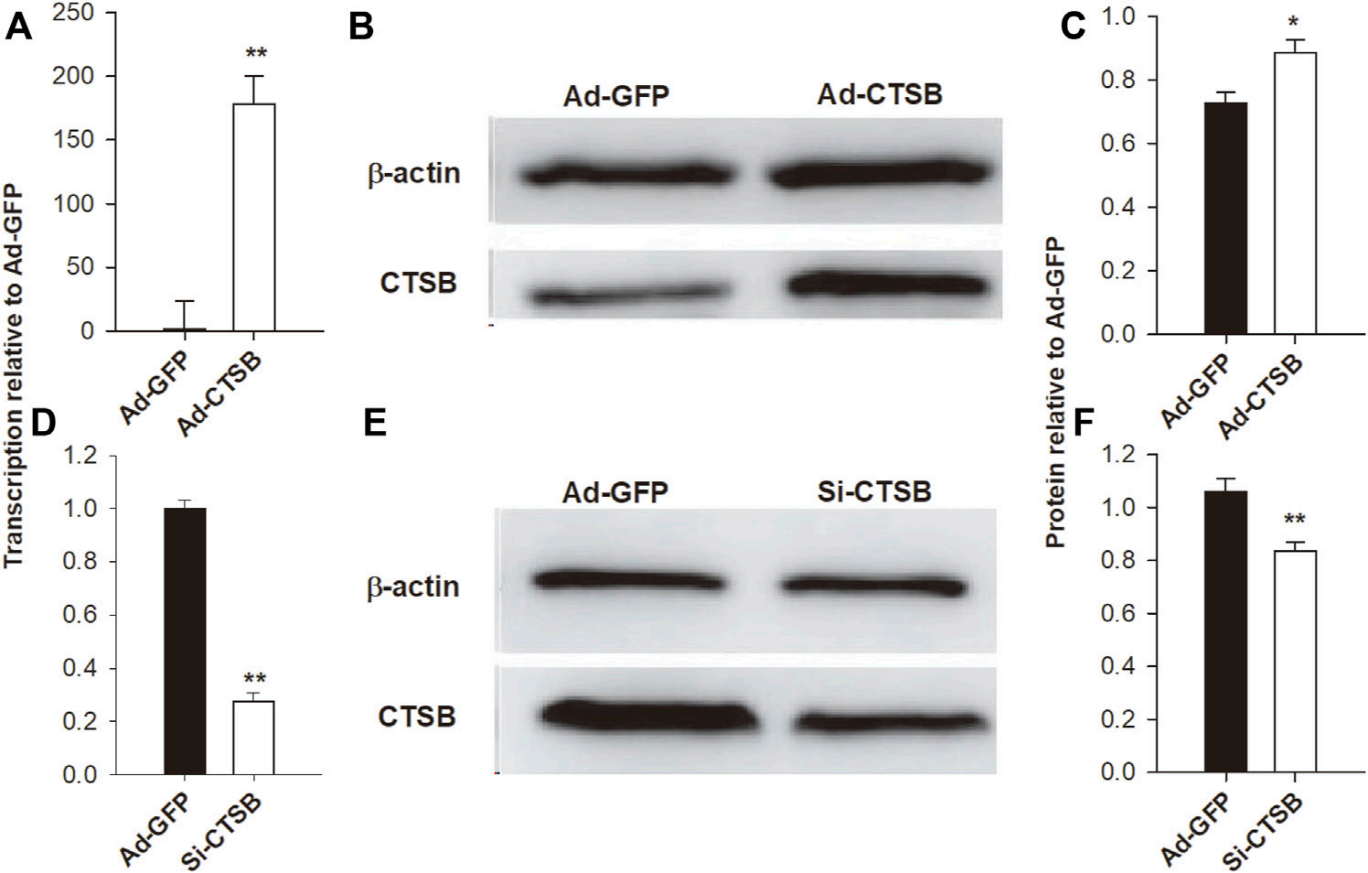
Adenovirus-mediated overexpression and siRNA-mediated interference of *CTSB* in rabbit GCS. The transcription of mRNA *via* RT-qPCR **(A,D)** and the protein expression *via* Western blot **(B,C,E,F)** of CTSB were highly affected by overexpression and interference treatments. Each experiment was performed with three biological replicates, and the results are expressed as the mean ± SEM. *p < 0.05, **p < 0.01.

To study the role of CTSB in GCS, we overexpressed and silenced the gene. The transcription and protein expression of *CTSB* were significantly affected by overexpression (Ad-CTSB) and silencing (siRNA) (Figure 5).

### CTSB Accelerates the Proliferation Activity in Rabbit GCS

**FIGURE 6:**
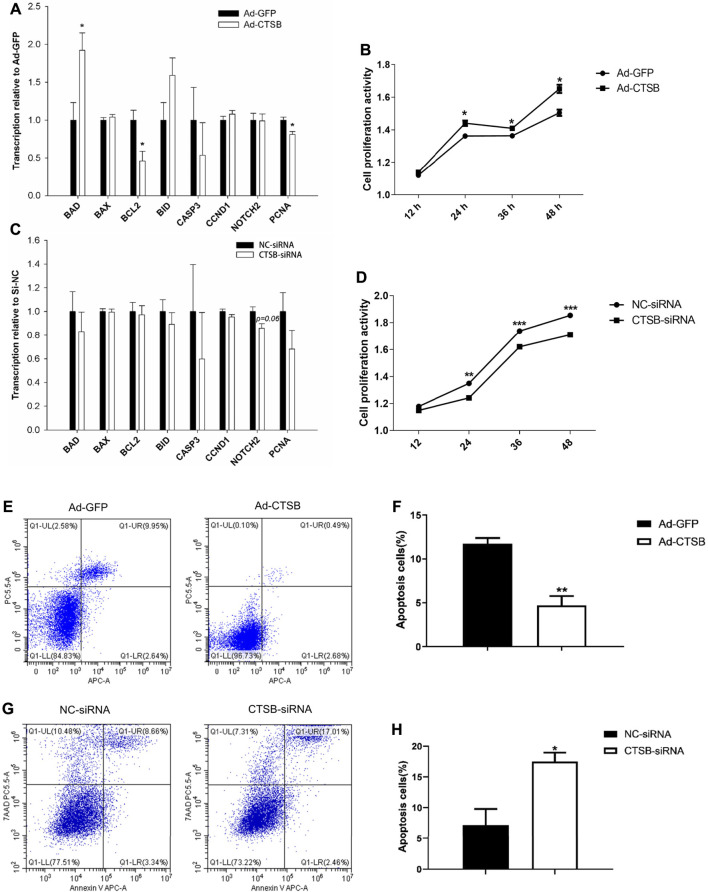
CTSB overexpression and interference on the proliferation and apoptosis of rabbit GCS. Transcription of genes associated with cell proliferation and apoptosis after CTSB overexpression **(A)** and knockdown **(C)**. Rabbit GCS proliferation after CTSB overexpression **(B)** and knockdown **(D)**. CTSB overexpression decreased the apoptosis in GCS as assessed by flow cytometer **(E,F)**. CTSB knockdown increased apoptosis of GCS as assessed by using a flow cytometer **(G,H)**. *p < 0.05; **p < 0.01.

Transcription of proliferating cell nuclear antigen (*PCNA*), a marker of cell proliferation, was significantly decreased (*p* < 0.05) by *CTSB* overexpression (Figure 6A) and numerically decreased with *CTSB* knockdown (Figure 6C). While transcription of the other two proliferation related genes, *CCND1* and *NOTCH2*, was not affected by CTSB overexpression (*p* > 0.05) but *NOTCH2* tended (*p* = 0.06) to be reduced by *CTSB* knockdown.

In spite of no important changes in genes associated with cell proliferation, overexpression or inhibition of *CTSB* strongly affected proliferation when measured at 12, 24, 36, and 48 h after insertion of Ad-CTSB or CTSB-siRNA (Figure 6B), with greater cell proliferation activity by CTSB overexpression (*p* < 0.05), while CTSB interference decreased cell proliferation activity (Figure 6D, *p* < 0.05).

### CTSB Decreased Cell Apoptosis in Rabbit GCS

Compared with the Ad-GFP group, the mRNA level of the main anti-apoptotic gene, *BCL2*, was decreased, while the pro-apoptotic gene, *BAD*, was increased by *CTSB* overexpression (Figure 6B, *p* < 0.05). Transcription of genes involved in apoptosis was not affected by silencing *CTSB*. Despite the transcription of apoptosis-related genes indicated no effect by silencing and minor effect by the overexpression of *CTSB*; flow cytometry analysis revealed that *CTSB* overexpression inhibits apoptosis of rabbit GCS (Figures 6E,F, *p* < 0.01). As shown in Figures 6G,H, *CTSB* knockdown led to a remarkable increase of apoptosis in rabbit GCS (*p* < 0.05).

### CTSB Promotes Secretion of Progesterone and Estradiol in Rabbit GCS

**FIGURE 7:**
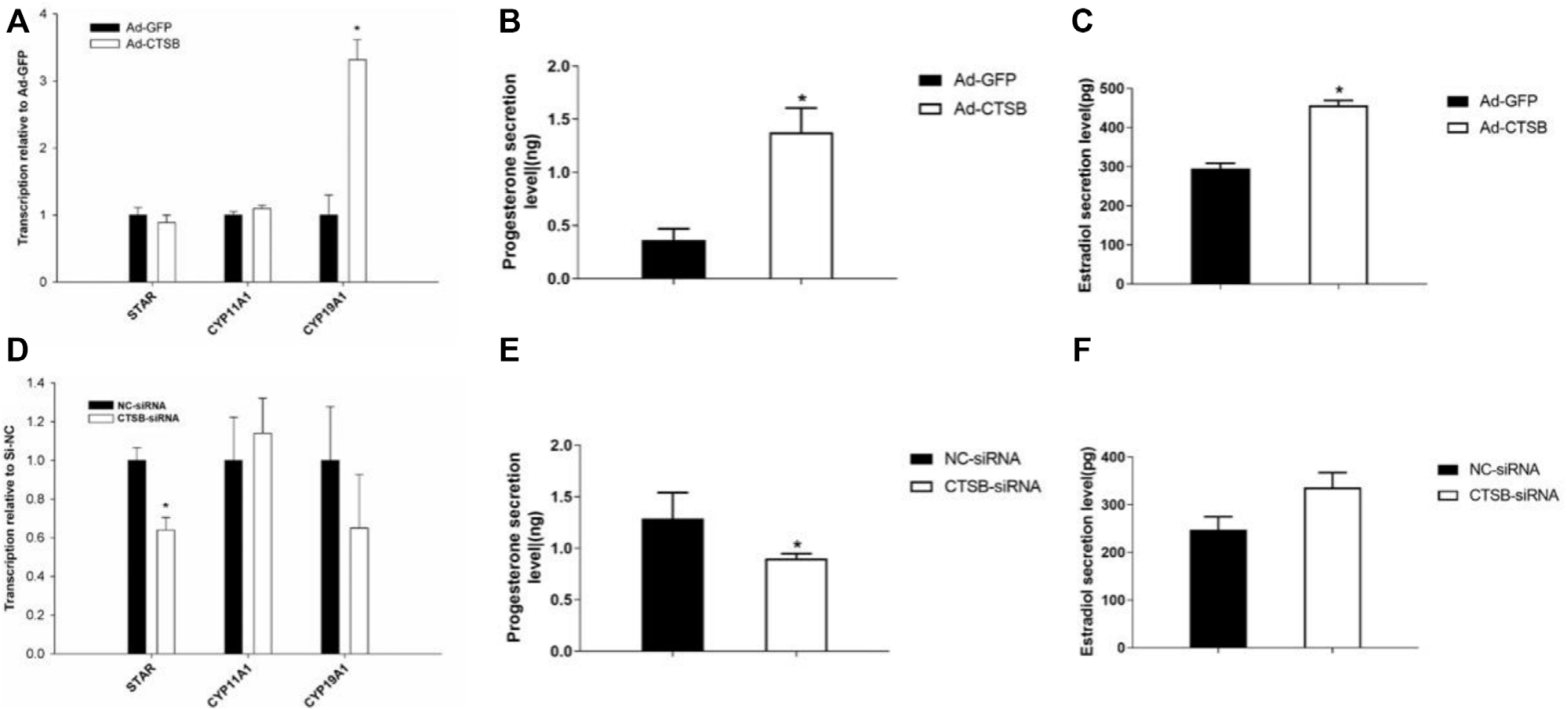
Effects of CTSB overexpression and interference on progesterone and estradiol secretion in rabbit GCS. Transcription of key genes related to hormone secretion in GCS with CTSB overexpression **(A)** or interference **(D)**. Level of progesterone and estradiol secretion in rabbit GCS after CTSB overexpression **(B,C)** and interference **(E,F)**. Each experiment had three biological replicates and the results are expressed as the mean ± SE. * *p* < 0.05, ** *p* < 0.01.

In order to further explore the function of the CTSB gene in reproduction and the potential link between this gene and the reduced fertility when coprophagy was prevented, we measured the effects of CTSB on the mRNA expression of genes related to progesterone and estradiol secretion. Transcription of *CYP19A1* was significantly upregulated by *CTSB* overexpression (Figure 7A, *p* < 0.05), together with the significant increase of progesterone and estradiol secretion (Figures 7B,C, *p* < 0.05). CTSB knockdown decreased the mRNA expression of steroidogenic acute regulatory protein (*STAR*) and progesterone level (Figures 7D,E, *p* < 0.05), while the estradiol level was not affected (Figure 7F, *p* > 0.05).

## Discussion

In our study we used the Elizabeth circle to establish a coprophagy prevention model of rabbits (Hughes and Gorospe, 1991). We found that preventing coprophagy did not cause any difference in food intake, but the body weight was decreased. This phenomenon may be caused by change in the intestinal microbiota and food digestion (McBee, 1971; Naumova et al., 2015).

As hypothesized, preventing coprophagy drastically affected reproduction by both decreasing progesterone secretion and inducing 100% fetal death in pregnant rabbits. Coprophagy is important for maintaining the normal reproductive performance, mainly because of the nutritional value of soft feces. However, the present study demonstrated that the failure to give birth to live pups was partly due to an increased apoptosis of ovaries, especially in granulosa cells. This is an important finding, since granulosa cells play an important role in the reproductive process and are mainly involved in the secretion of progesterone and estrogen (Gore-Langton and Daniel, 1990; Cai et al., 2015). Estradiol and progesterone are important hormones that play a critical regulatory role in maintaining normal reproduction in mammals (Cerri et al., 2011; Martins et al., 2014), including development of follicles (Chang et al., 2016). Granulosa cells maintain healthy oocytes *via* transzonal projections (Li and Albertini, 2013) and apoptosis of granulosa cells affects oocytes directly (Emanuelli et al., 2019), also causing atresia of the follicles (Hughes and Gorospe, 1991; Tilly et al., 1991; Manabe et al., 2004). Overall, our data indicated an important role of coprophagy in maintaining normal reproduction performance of rabbits, but it is unclear the reason for such an effect.

In order to further reveal the mechanism of coprophagy on reproduction and specifically on the ovaries, we performed a whole transcriptome sequencing of RNA isolated from the ovaries. The results indicate a very modest effect of coprophagy on ovary transcriptome but revealed a downregulation of pathways involved in lipid metabolism, immune response, cell proliferation, and organelles involved with apoptosis, such as lysosomes. Based on this, the *CTSB* gene which was significantly decreased in expression by coprophagy appeared to be an important hub, suggesting a possible role in connecting the prevention of coprophagy with reproductive failure.

As one member of the cathepsin family, CTSB is the most abundant lysosomal cysteine protease, which is generally considered as intracellular protease (Aggarwal and Sloane, 2014). It plays an important role in the catabolism of proteins in cells and participates in various physiological processes, including oxidative stress and inflammatory response (Bai et al., 2018; Lian et al., 2018). Moreover, CTSB is related to the development of the cumulus cells-oocyte complex, thereby affecting reproductive performance (Oksjoki et al., 2001; Wei et al., 2019). In our study, we detected the good expression of CTSB in rabbit ovarian tissue.

In order to identify the function of CTSB in the apoptosis of cumulus cells in rabbits, we manipulated the expression of the CTSB gene in primary cumulus cells. We found that the overexpression or underexpression of the CTSB gene promotes or inhibits the growth of GCS, respectively. Reduced cell proliferation was observed when the CTSB gene was inhibited in cholangiocarcinoma cells (Li et al., 2018). Among all genes associated with cell growth, overexpression of CTSB increased the expression of the pro-apoptotic gene *BAD* (Basu, 2021), while decreased the expression of anti-apoptotic BCL2 (Basu, 2021) and the PCNA which is important for cell proliferation (Zhang, 2021). Interference of *CTSB* expression did not affect transcription of any apoptosis-related genes. Those findings indicate that the effect of CTSB on apoptosis is not *via* change in the transcription of apoptosis-related genes. The effect on apoptosis by CTSB might be by protease activity toward pro-apoptotic proteins, as previously observed in human cells (Seng et al., 2016). Taken together, our results indicated that the expression of *CTSB* was decreased by preventing coprophagy in rabbits. The decrease of CTSB appears to be related to the increased apoptosis of GCS likely *via* reduced proteolysis of pro-apoptotic proteins affecting the reproductive performance of rabbits. The negative role of apoptosis of granulosa cells on reproduction is supported by a recent study, indicating that the apoptosis of granulosa cells can cause follicular atresia and decrease reproductive performance, as observed in pigs (Zhao et al., 2014; He et al., 2016). This effect was also confirmed during the process of cancer progression, in which CTSB deletion significantly increases tumor cell death (Gocheva et al., 2006).

`Interestingly, we also found that modulation of the CTSB gene had a great influence on the secretion of progesterone and estradiol in rabbit GCS. In this study, overexpression of the CTSB gene increased the secretion of sterol hormones (progesterone and estradiol) and the expression of *CYP19A1*, coding for the estrogen synthetase. The central role of CTSB on the decreased secretion of progesterone in rabbits that were not allowed to do coprophagy was demonstrated in our study by the manipulation of CTSB expression. Although CTSB gene interference resulted in decreased progesterone secretion, it did not affect estradiol secretion. However, the interference of *CTSB* expression did not affect estrogen synthetase but decreased the expression of the steroidogenic acute regulatory protein (STAR), a key gene for the transport of cholesterol into the mitochondria for the synthesis of pregnenolone, the main steroidogenic precursor (Schumacher et al., 2017).

In our study, we only evaluated the role of one of the 241 DEGs in ovary as consequence of preventing coprophagy. Thus, we cannot exclude that change in those other DEGs played a critical role; however, this need to be evaluated *via* molecular approaches as performed in the present manuscript. In addition, the link between CTSB and the change in expression of the few affected genes is still unclear, since CTSB is a protease.

## Conclusion

**FIGURE 8:**
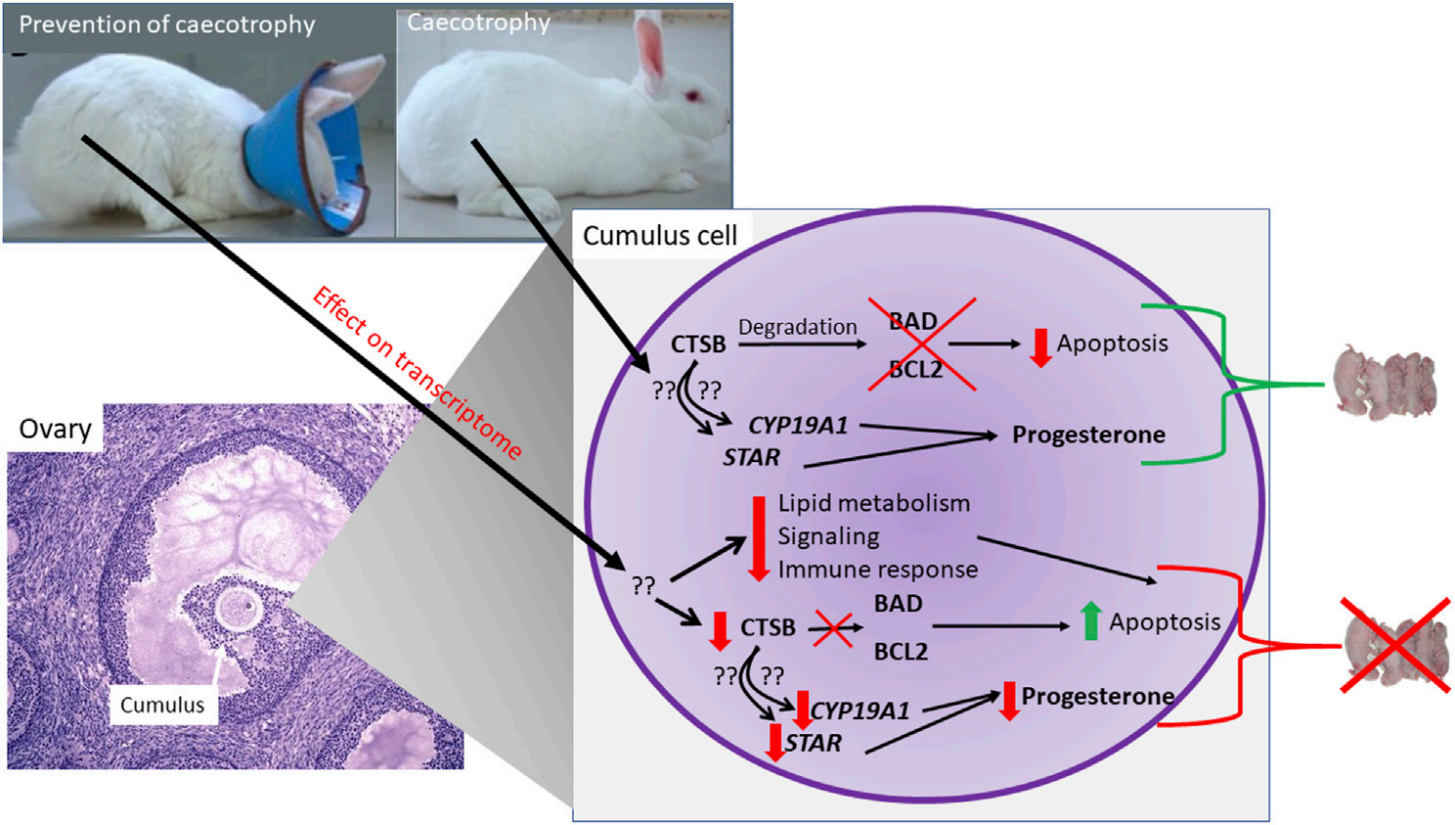
Model summarizing findings from the experiment.

In conclusion, our study demonstrated an important role of coprophagy on growth and reproductive performance of rabbits. Our data indicated an increased apoptosis of granulosa and a decrease secretion of progesterone when coprophagy was prevented. Our data revealed some effects on the transcriptome of the ovaries by preventing coprophagy with downregulation of genes associated with pathways related to cell growth, immune response, and metabolism. The transcriptome data together with the bioinformatic analyses allowed to identify CTSB as a potential central hub in the observed effects. The subsequent molecular studies on granulosa cells demonstrated a role of CTSB in controlling apoptosis and secretion of progesterone. The role of CTSB is not *via* change in expression of genes but likely *via* protease activity on apoptosis-related proteins. In Figure 8 we summarized our findings.

## Data Availability

The datasets presented in this study can be found in online repositories. The names of the repository/repositories and accession number(s) can be found in the following link: https://www.ncbi.nlm.nih.gov/, PRJNA767495.
