# Supplementary material for: Coprophagy Prevention Decreases the Reproductive Performance and Granulosa Cell Apoptosis via Regulation of CTSB Gene in Rabbits
Source: Front Physiol. 2022 Jul 18;13:926795. doi: 10.3389/fphys.2022.926795 (PMC9341522; doi:10.3389/fphys.2022.926795)
Supplement: Supplementary file 1 [file DataSheet3.docx]

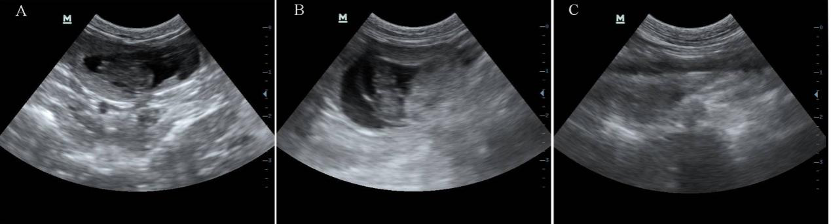


**FIGURE S1.** Visualization of rabbit fetal by B-ultrasound at 15 days of pregnancy. **A:** Control group; **B:** Experimental group. **C:** Blank group (non-pregnant rabbit).


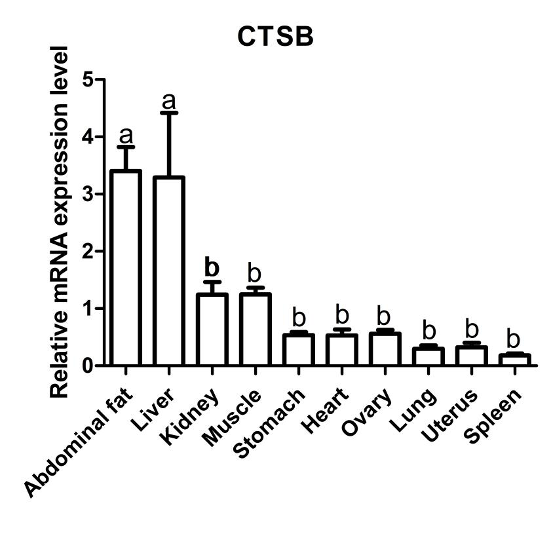


**FIGURE S2.** Transcriptional level of CTSB gene in different organs and tissues in rabbits.


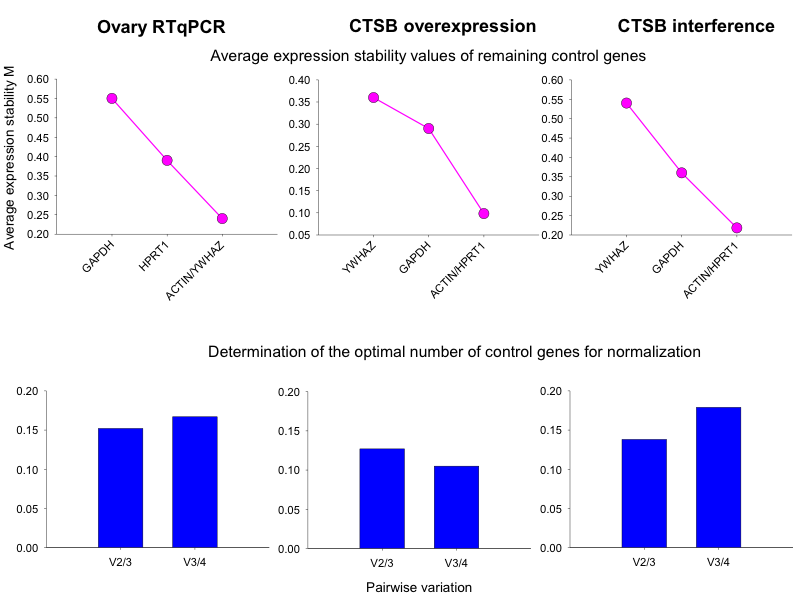


**FIGURE S3.** geNorm results from the assessment of reference genes.

**TABLE S1.** Name, accession number, sequences, amplicon length of primer pairs used in the present experiment.

| **Gene name** | **Symbol/Acc#** | **Primers** | **Sequence(5' to 3')** | **bp** |
| --- | --- | --- | --- | --- |
| BCL2 Associated Agonist of Cell Death | *BAD* | F.436 | CGAAGGATGAGCGACGAGTT | 144 |
|  | XM_002724450.3 | R.579 | GCCTCCTTTCCCCAAATTGC |  |
| BCL2 Associated X, Apoptosis Regulator | *BAX* | F.11 | CCGGGGAGCAGTCCAGA | 167 |
|  | XM_002723696.3 | R.177 | CAGCTTCTTGGTGGACTCGT |  |
| B-cell lymphoma 2 | *BCL2* | F.492 | GACTGAGTACCTGAACCGGC | 166 |
|  | XM_008261439.2 | R.65 | GAGGGTGATGCAAGCTCCTA |  |
| BCL2-like protein 1 | *BCL2L1* | F.449 | CACCCCAGGGACAGCATATC | 160 |
|  | XM_008256141.2 | R.608 | CGCGATCCGACTCACCAATA |  |
| BCL-2-like protein 2 | *BCL2L2* | F.369 | CGCTTCCGGCAAAACTTCTC | 173 |
|  | XM_008269377.2 | R.541 | TTGTTGACGCTCTCAGCACA |  |
| BH3 Interacting Domain Death Agonist | *BID* | F.415 | GATGACGAACTGCAGACGGA | 226 |
|  | XM_017338485.1 | R.640 | TGTGGCCATCCATTCACTCC |  |
| Bone Morphogenetic Protein 2 | *BMP2* | F.483 | TCCCCCGGAGGAGTTTATCA | 91 |
|  | NM_001082650.1 | R.573 | ATGATGGAAACCGCTGTCGT |  |
| Caspase 3 | *CASP3* | F.1 | ATGGAGAACAACGAAACCTCC | 191 |
|  | NM_001082117.1 | R.191 | CGGGACGACATTCCAGTGTT |  |
| Caspase 6 | *CASP6* | F.175 | GGCACTAATGCAGACCGAGA | 271 |
|  | XM_017347353.1 | R.445 | TGGGTTTTCCAACCAGGCTT |  |
| Cyclin D1 gene | *CCND1* | F.44 | GCAGCCCTTTCAATGCTGAC | 244 |
|  | XM_017348091.1 | R.697 | CTTTGGACGCTCTGACCAGT |  |
| CD36 Molecule | *CD36* | F.306 | AACTGTGGTCTTATCGCGGG | 162 |
|  | XM_008258301.2 | R.467 | TGTGCCTGTTTTCACCCAGT |  |
| CD9 Molecule | *CD9* | F.12 | GGTGCTCAGAACTAACGCCT | 94 |
|  | XM_017343606.1 | R.105 | CTGAGAGTCGAATCGGAGCC |  |
| Cathepsin B | *CTSB* | F.208 | CGCAGAGTTGAGTTCGCTGA | 107 |
|  | XM_002709436.3 | R.314 | CAGGACCCCTGGTCTCTGAT |  |
|  | XM_002709436.3 | R.1142 | ACAGCGGGTTAGATCTGTCG |  |
| Cathepsin S | *CTSS* | F.862 | GGCGTACTAGCAGTTGGCTAT | 73 |
|  | XM_002715580.3 | R.934 | GGATGCCCCAGCTGTTTTTC |  |
| Cytochrome P450 Family 11 Subfamily A Member 1 | *CYP11A1* | F.1259 | GCCGGGAACCTAGCTTCTTT | 155 |
|  | XM_008253734.2 | R.1413 | GATGCTCATCTCCACCTCGG |  |
| Cytochrome P450 Family 19 Subfamily A Member 1 | *CYP19A1* | F.463 | CCTGGGCTTGTTCAGATGGT | 171 |
|  | NM_001170921.2 | R.633 | ACTTTCGTCCATGGGGATGC |  |
| Glyceraldehyde-3-phosphate dehydrogenase | *GAPDH* | F.80 | GAAGGTCGGAGTGAACGGAT | 212 |
|  | NM_001082253.1 | R.291 | TGATGGCCTTCCCGTTGATG |  |
| Growth differentiation factor-9 | *GDF9* | F.115 | GCGTTGGAGTCTGAGGTTGA | 190 |
|  | NM_001171350.1 | R.304 | TCCCCTCCTTGGTAGCGTAA |  |
| Glycoprotein Nmb | *GPNMB* | F.1299 | TCTCTGTCCCTGGTAGAGCC | 113 |
|  | XM_008261526.2 | E.1411 | TCTGTACACGTAGAGGGCGA |  |
| Integrative and Conjugative Elements | *ICE* | F.5959 | CAAAGCCATAGCCGATGCAC | 243 |
|  | XM_008251200.1 | R.6201 | AGGGCGTGGATGTAACTGTG |  |
| Lipoprotein Lipase | *LPL* | F.1087 | TCAACCCGGGTGTAACATCG | 186 |
|  | NM_001177330.1 | R.1272 | AGCCCCTTCTCAAAGGCTTC |  |
| Induced myeloid leukemia cell differentiation protein | *MCL1* | F.599 | GCCAGTAAGGTCCCTAGCAC | 98 |
|  | XM_002715585.3 | R.696 | ATAATCTCCAGCGACTGCCG |  |
| NADP-dependent malic enzyme | *ME1* | F.1617 | CTGGCCAAGGCAACAATTCC | 164 |
|  | XM_002714552.3 | R.1780 | CAGGGGAGGATAAAGTCGGC |  |
| Notch Receptor 2 | *NOTCH2* | F.3798 | CAACCGCCAGTGTGTTCAAG | 232 |
|  | XM_017345939.1 | R.4029 | CTTCCGCTTTCGTTTTGCCA |  |
| Proliferating Cell Nuclear Antigen | *PCNA* | F.432 | TGCACGTATATGCCGAGACC | 240 |
|  | XM_017341762.1 | R.671 | GTAGGAGAAAGCGGAGTGGC |  |
| Steroidogenic Acute Regulatory Protein | *STAR* | F.477 | GATTGGGAAGGACACGGTCA | 179 |
|  | XM_017350353.1 | R.655 | CACCCCTGATGACGCCTTT |  |
