# Supplementary material for: Coprophagy Prevention Decreases the Reproductive Performance and Granulosa Cell Apoptosis via Regulation of CTSB Gene in Rabbits
Source: Front Physiol. 2022 Jul 18;13:926795. doi: 10.3389/fphys.2022.926795 (PMC9341522; doi:10.3389/fphys.2022.926795)
Supplement: Supplementary file 2 [file DataSheet1.ZIP › Original data/Figure 5 Western blot result/siRNA-mediated interference/Statement.docx]

**Statement**

siRNA-mediated interference of CTSB in rabbit GCS，From left to right are siRNA-mediated interference group，control group，siRNA-mediated interference group，control group，siRNA-mediated interference，control group，The pictures in this experiment are the first and second lanes.
