# Supplementary material for: Coprophagy Prevention Decreases the Reproductive Performance and Granulosa Cell Apoptosis via Regulation of CTSB Gene in Rabbits
Source: Front Physiol. 2022 Jul 18;13:926795. doi: 10.3389/fphys.2022.926795 (PMC9341522; doi:10.3389/fphys.2022.926795)

CTSB overexpression and interference on the apoptosis of rabbit GCS, Each experiment was performed with 3 biological replicates. The pictures in this article are picture NC-siRNA-1

，CTSB-siRNA-2 and picture Ad-GFP-1，Ad-GFP-1.

NC-siRNA-1


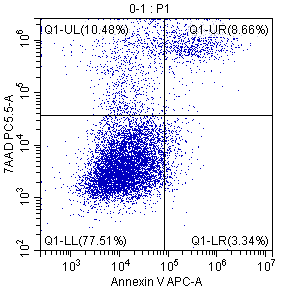


NC-siRNA-2


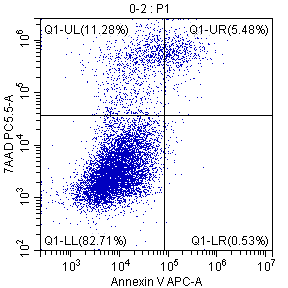


NC-siRNA-3


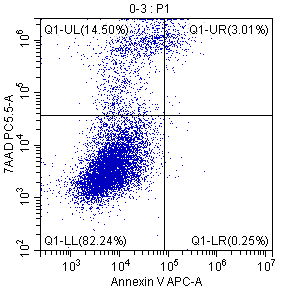


CTSB-siRNA-1


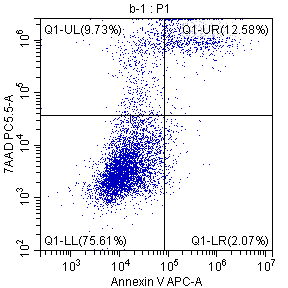


CTSB-siRNA-2


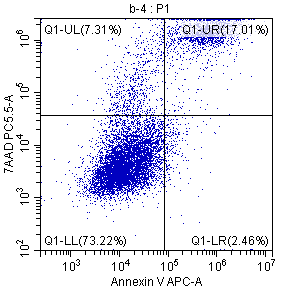


CTSB-siRNA-3


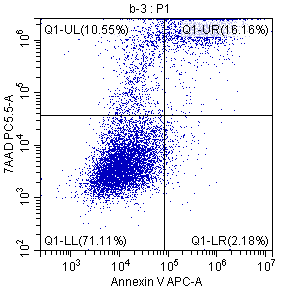


Ad-GFP-1


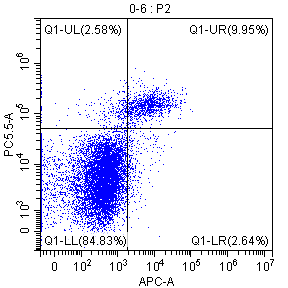


Ad-GFP-2


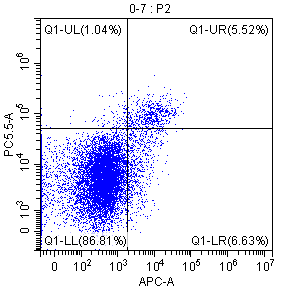


Ad-GFP-3

Ad-CTSB-1


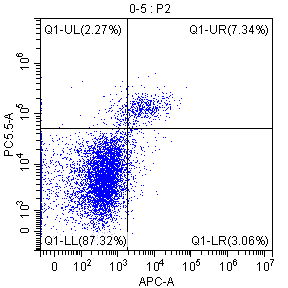


Ad-CTSB-2


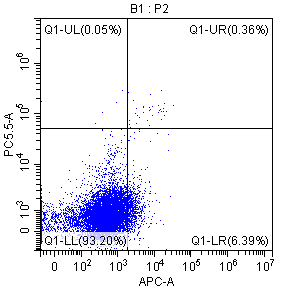


Ad-CTSB-3


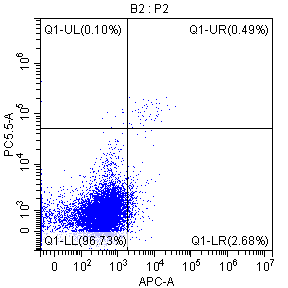

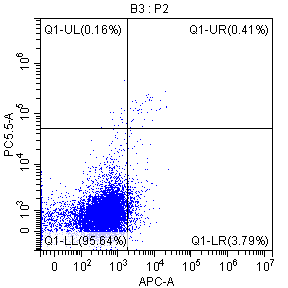

Supplement: Supplementary file 2 [file DataSheet1.ZIP › Original data/Figure6/Flow cytomete date.docx]
