# Supplementary figures and images for: Coprophagy Prevention Decreases the Reproductive Performance and Granulosa Cell Apoptosis via Regulation of CTSB Gene in Rabbits
Source: Front Physiol. 2022 Jul 18;13:926795. doi: 10.3389/fphys.2022.926795 (PMC9341522; doi:10.3389/fphys.2022.926795)

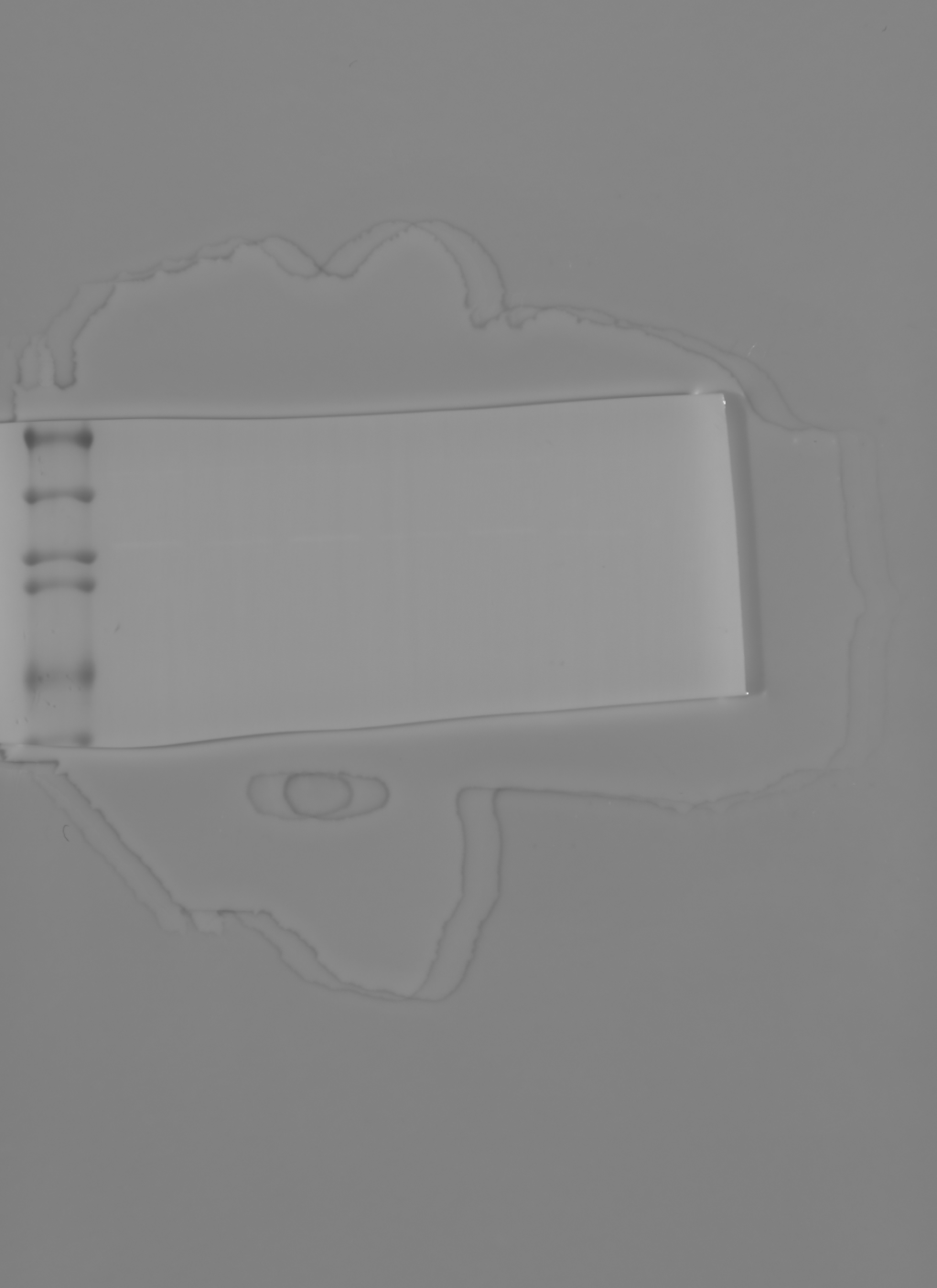

Supplement: Supplementary file 2 [file DataSheet1.ZIP › Original data/Figure 5 Western blot result/overexpression/overexpression-Marker.tif]

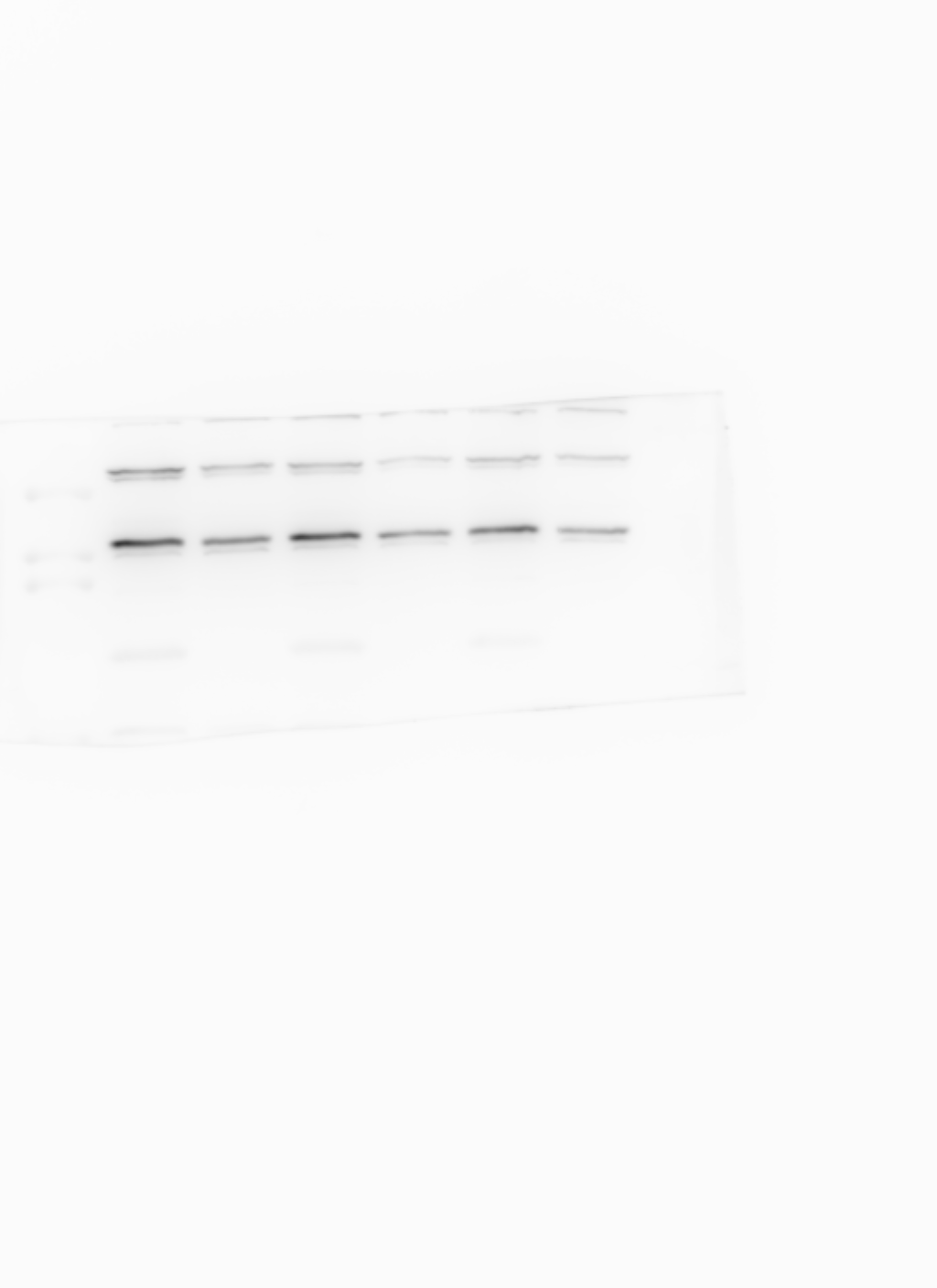

Supplement: Supplementary file 2 [file DataSheet1.ZIP › Original data/Figure 5 Western blot result/overexpression/overexpression.tif]

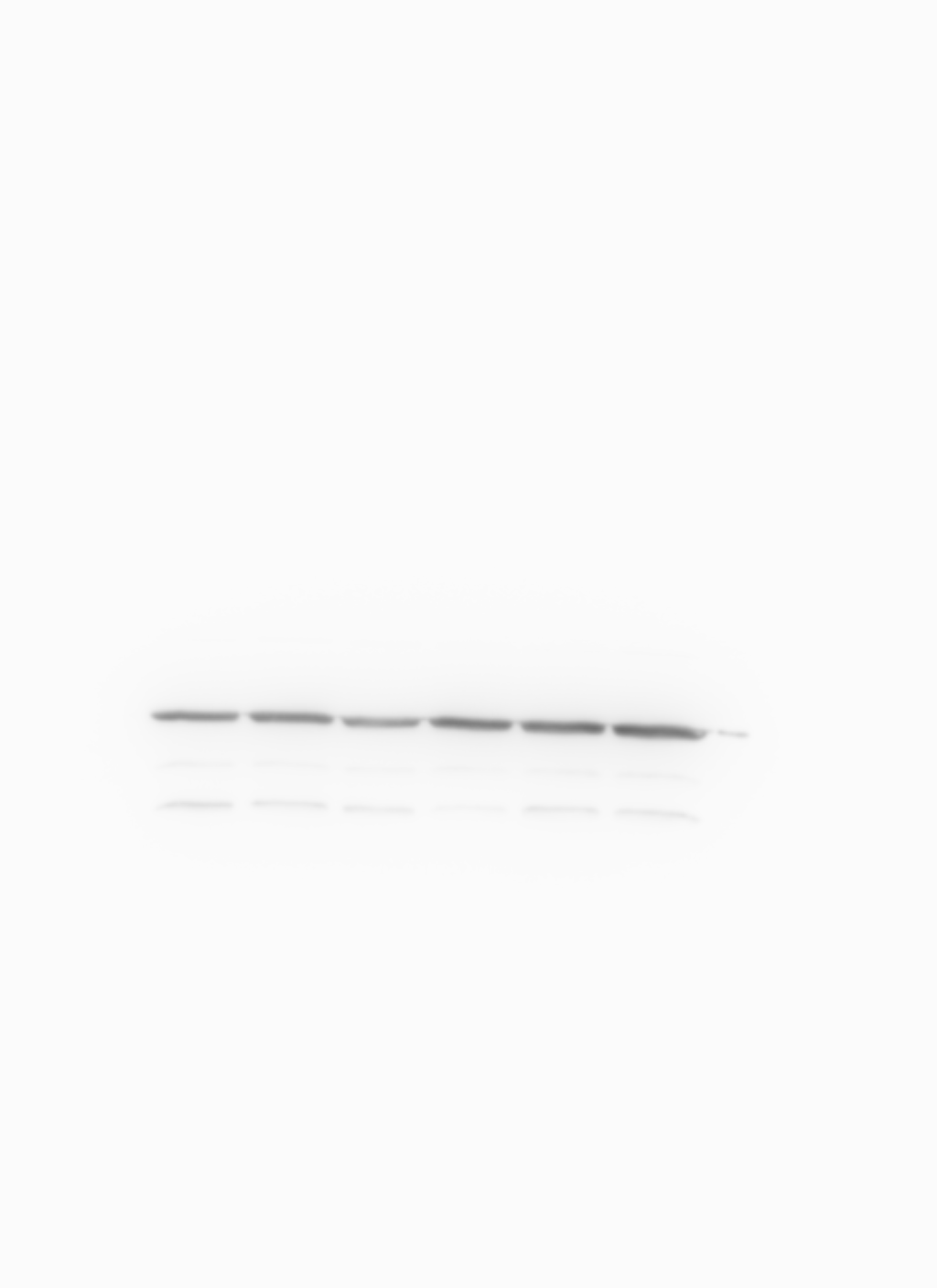

Supplement: Supplementary file 2 [file DataSheet1.ZIP › Original data/Figure 5 Western blot result/siRNA-mediated interference/actin.tif]

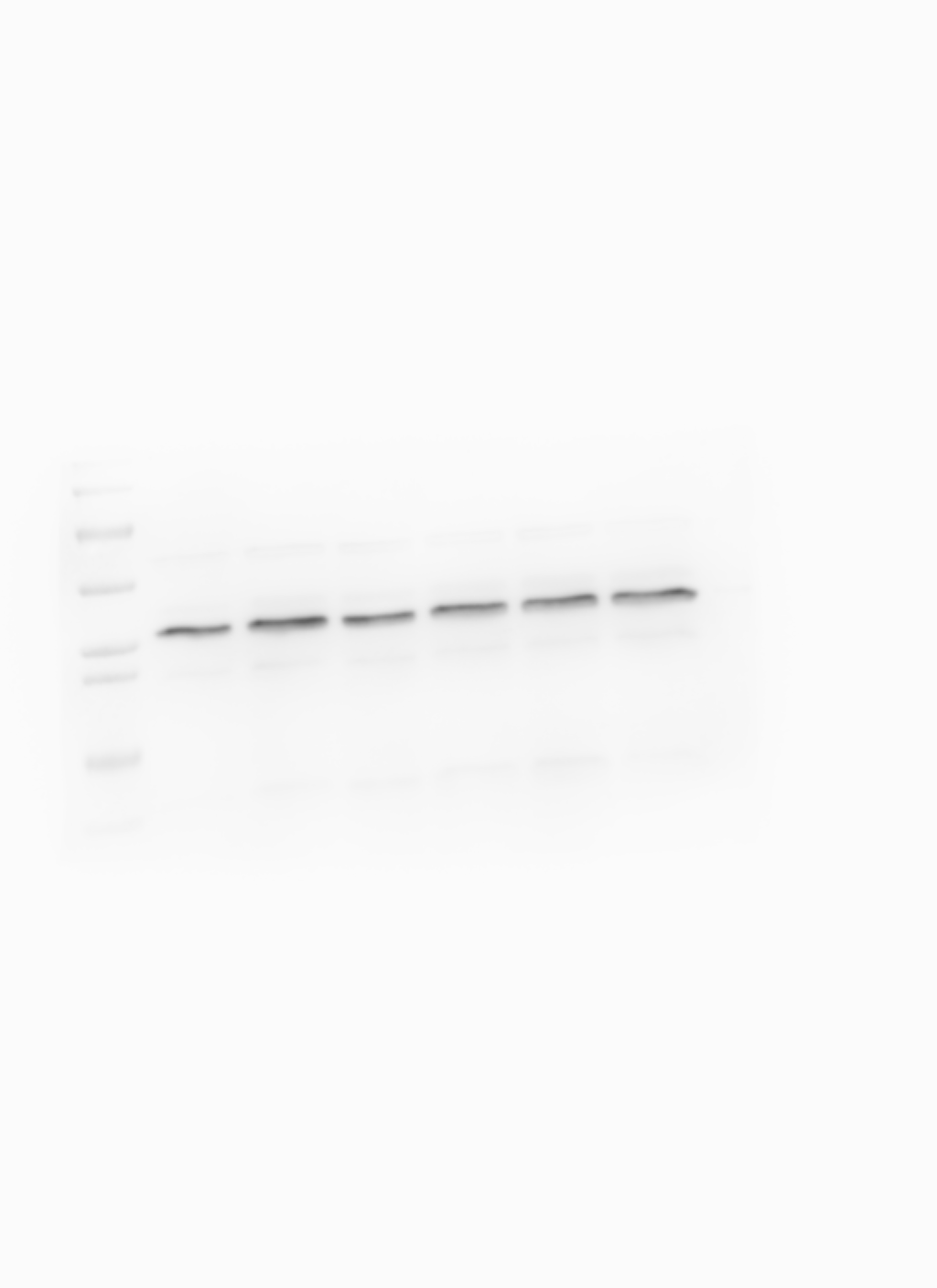

Supplement: Supplementary file 2 [file DataSheet1.ZIP › Original data/Figure 5 Western blot result/siRNA-mediated interference/CTSB.tif]

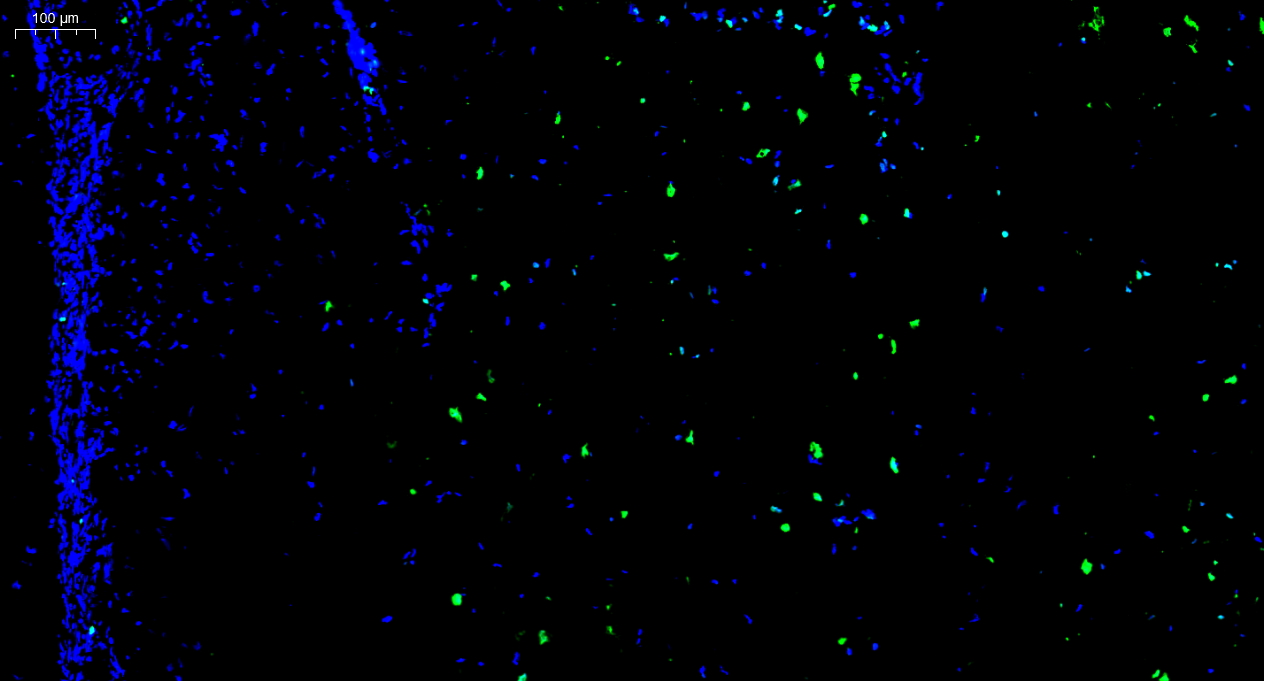

Supplement: Supplementary file 2 [file DataSheet1.ZIP › Original data/Figure2-cell apoptosis figure in ovary of rabbits/control group merge.jpg]

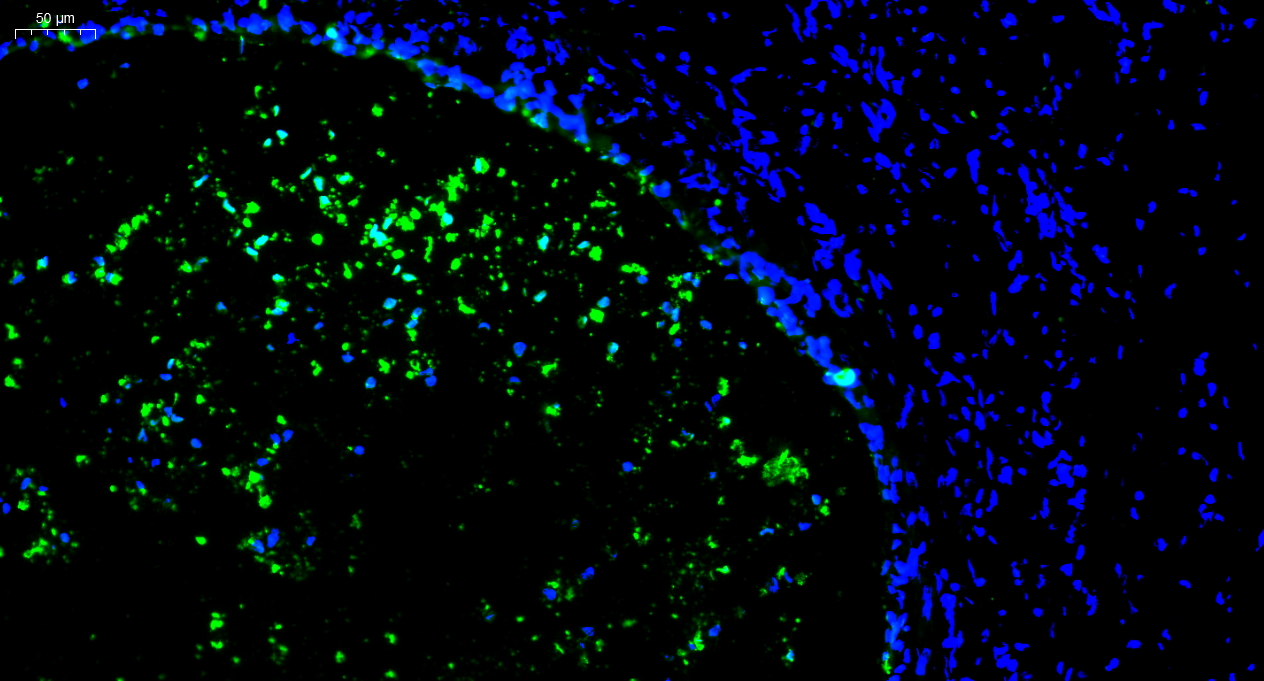

Supplement: Supplementary file 2 [file DataSheet1.ZIP › Original data/Figure2-cell apoptosis figure in ovary of rabbits/Experimentel group merge.jpg]

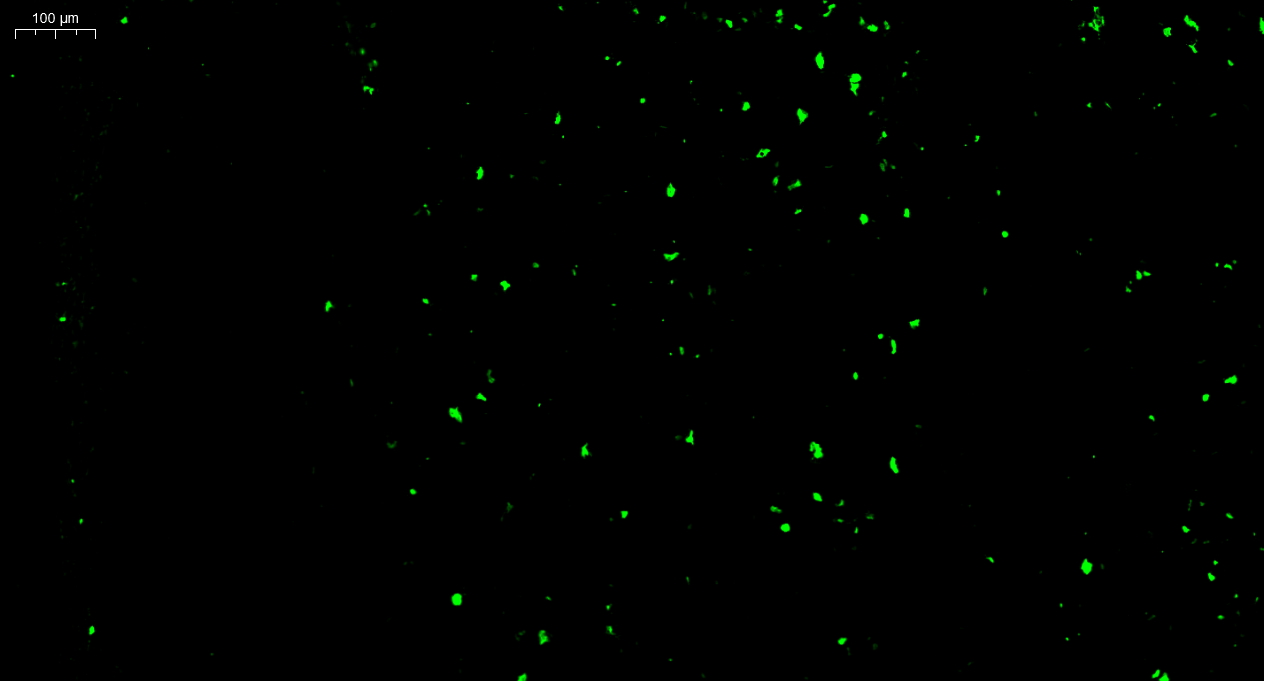

Supplement: Supplementary file 2 [file DataSheet1.ZIP › Original data/Figure2-cell apoptosis figure in ovary of rabbits/control group TUNEL (green).jpg]

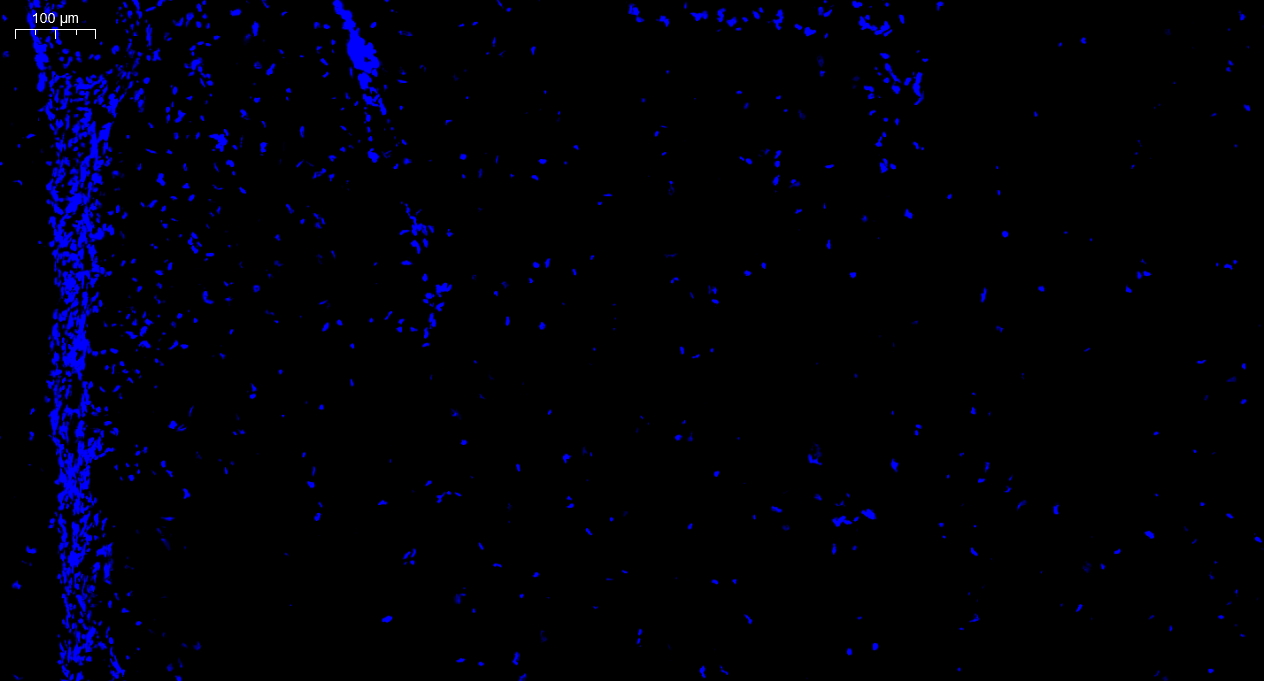

Supplement: Supplementary file 2 [file DataSheet1.ZIP › Original data/Figure2-cell apoptosis figure in ovary of rabbits/control group DAPI (blue).jpg]

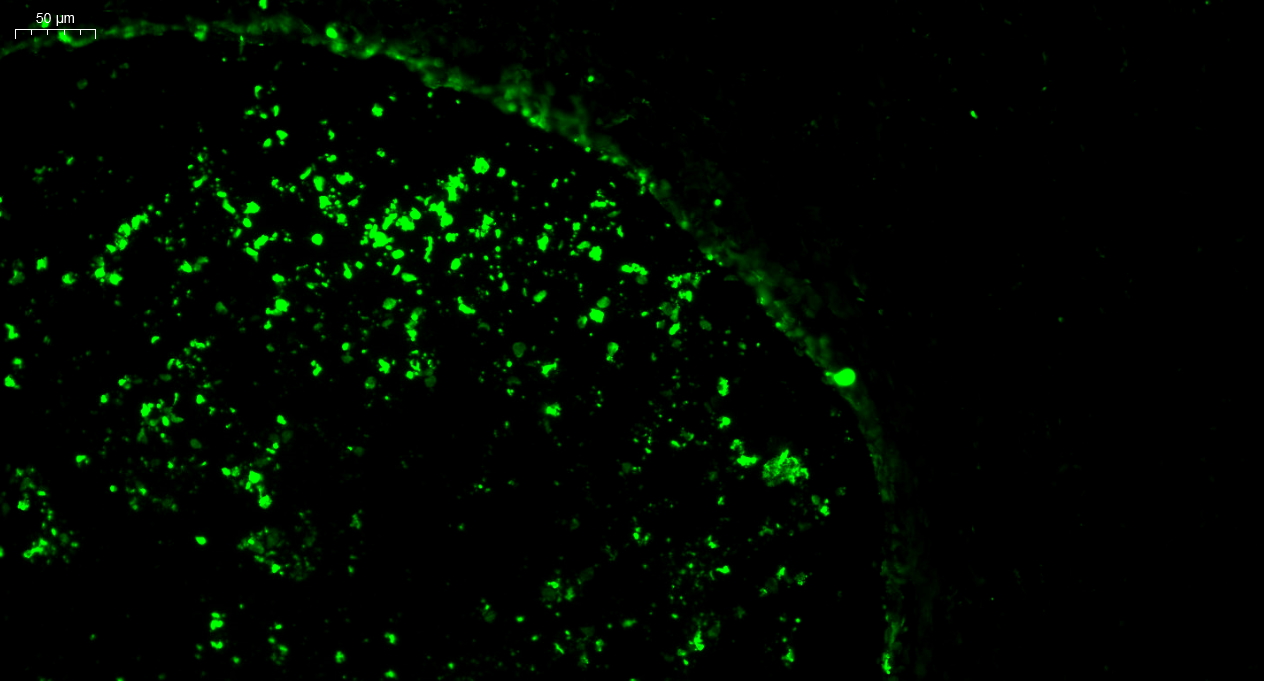

Supplement: Supplementary file 2 [file DataSheet1.ZIP › Original data/Figure2-cell apoptosis figure in ovary of rabbits/Experimentel group TUNEL (green).jpg]

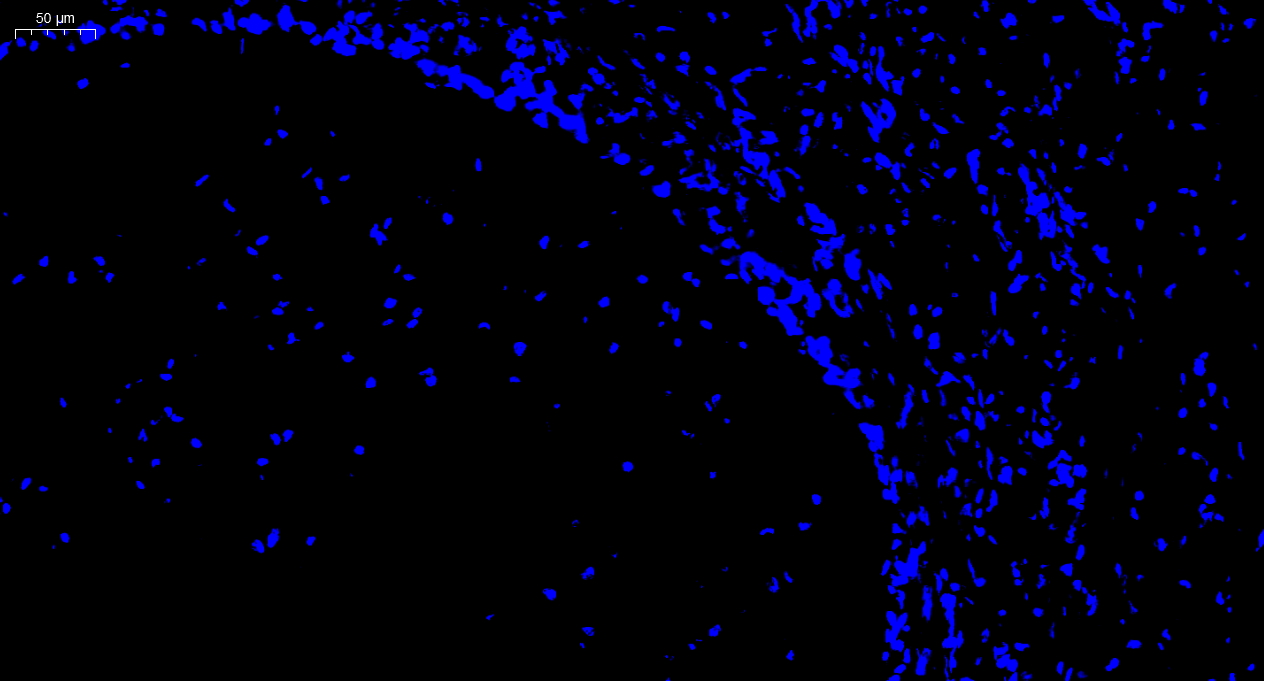

Supplement: Supplementary file 2 [file DataSheet1.ZIP › Original data/Figure2-cell apoptosis figure in ovary of rabbits/Experimentel group DAPI (blue).jpg]

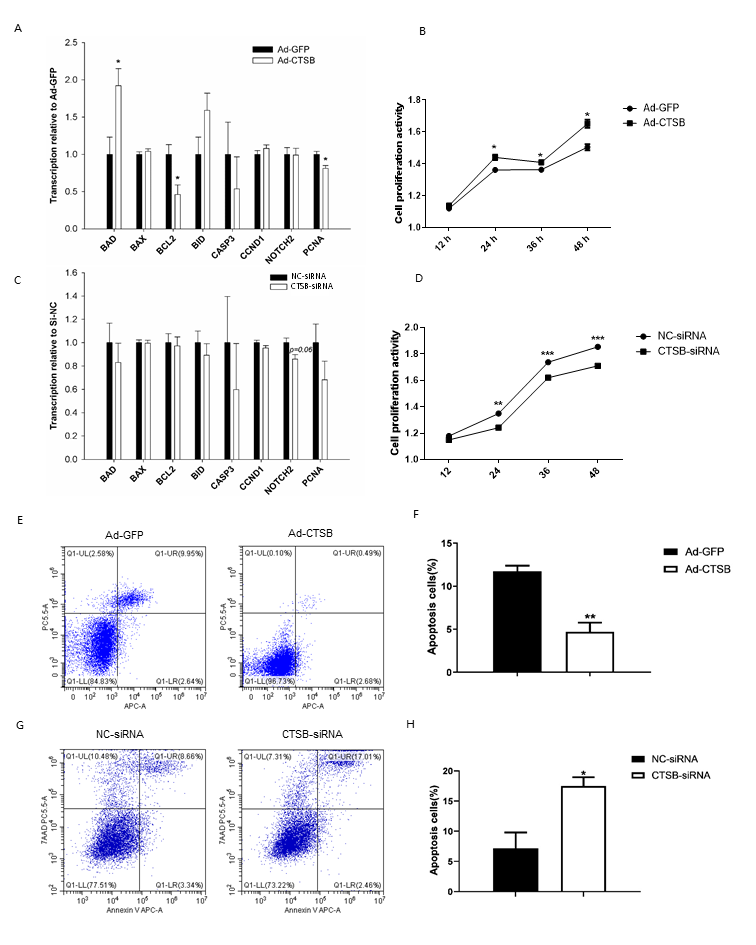

Supplement: Supplementary file 2 [file DataSheet1.ZIP › Original data/Figure6/σçïΣ║í+σó₧σÇ╝.png]

## Slide 1
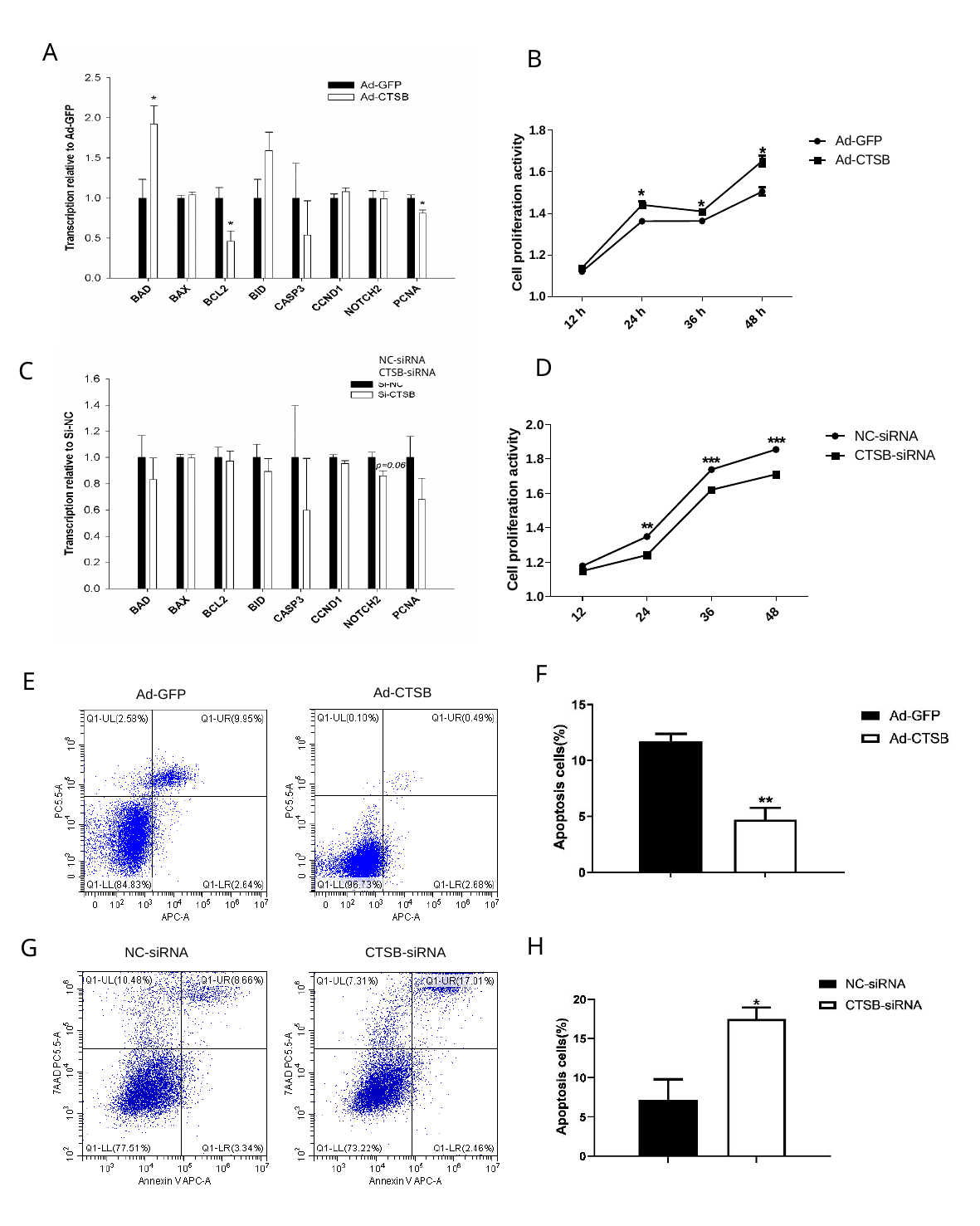

A
B
D
NC-siRNA
CTSB-siRNA
C
F
E
Ad-CTSB
Ad-GFP
H
G
CTSB-siRNA
NC-siRNA

Supplement: Supplementary file 2 [file DataSheet1.ZIP › Original data/Figure6/σçïΣ║í+σó₧σÇ╝.pptx]
