# Supplementary material for: Coprophagy Prevention Decreases the Reproductive Performance and Granulosa Cell Apoptosis via Regulation of CTSB Gene in Rabbits
Source: Front Physiol. 2022 Jul 18;13:926795. doi: 10.3389/fphys.2022.926795 (PMC9341522; doi:10.3389/fphys.2022.926795)
Supplement: Supplementary file 3 [file DataSheet2.ZIP › Raw data of flow experiment (For review purpose only)/Gating strategies.docx]

**1. Gating strategies for CTSB overexpression experiment**

Since the overexpression recombinant adenovirus of CTSB (Ad-CTSB) carrys GFP, we first built a forward scatter (FSC) vs side scatter (SSC) dot plot to exclude cellular debris and mutual adhesion cells by thresholding, and circled the cell population as the P1 gate. On the basis of the P1 gate, a FITC vs FSC dot plot was established, and the positive cell population was circled as the P2 gate (the granulosa cells that were successfully infected with adenovirus were screened). Then on the basis of the P2 gate, Annexin V/Alexa Fluor 647 and 7-AAD were stained by single staining. Blank tube and single positive tube were used to adjust voltage compensation, and finally got the flow data after adjusting compensation.

**2. Gating strategies for interference experiments**

Firstly, we built a forward scatter (FSC) vs side scatter (SSC) dot plot, excluded cell debris and adherent cells by thresholding, and circled the cell population as the P1 gate. On the basis of the P1 gate, Annexin V/Alexa Fluor 647 and 7-AAD were stained by single staining. After adjusting the voltage compensation with a blank tube and a single positive tube, the flow data were obtained.
